# Mesoporous Cubic Nanocages Assembled by Coupled Monolayers With 100% Theoretical Capacity and Robust Cycling

**DOI:** 10.1021/acscentsci.4c00345

**Published:** 2024-06-10

**Authors:** Guangtao Zan, Shanqing Li, Ping Chen, Kangze Dong, Qingsheng Wu, Tong Wu

**Affiliations:** † School of Chemical Science and Engineering, Institute of Advanced Study, Shanghai Key Laboratory of Chemical Assessment and Sustainability, Tongji University, Shanghai 200092, PR China; ¶ Department of Materials Science and Engineering, Yonsei University, Seoul 03722, Republic of Korea; § School of Materials and Environmental Engineering, Chizhou University, Chizhou, Anhui 247000, PR China; ∇ School of Chemistry and Chemical Engineering, Anhui University, Hefei, Anhui 230601, PR China

## Abstract

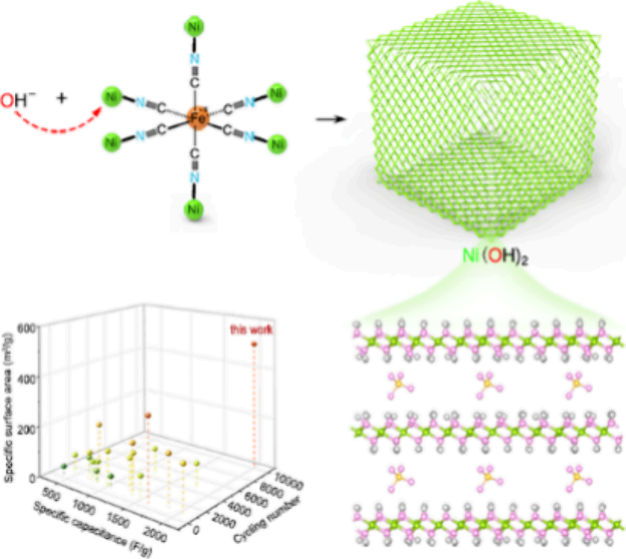

High capacity and long cycling often conflict with each other in electrode materials. Despite extensive efforts in structural design, it remains challenging to simultaneously achieve dual high electrochemical properties. In this study, we prepared brand-new completely uniform mesoporous cubic-cages assembled by large *d*-spacing Ni(OH)_2_ coupled monolayers intercalated with VO_4_^3–^ (NiCMCs) using a biomimetic approach. Such unique mesoporous structural configuration results in an almost full atomic exposure with an amazing specific surface area of 505 m^2^/g and atomic utilization efficiency close to the theoretical limit, which is the highest value and far surpasses all of the reported Ni(OH)_2_. Thus, a breakthrough in simultaneously attaining high capacity approaching the 100% theoretical value and robust cycling of 10,000 cycles is achieved, setting a new precedent in achieving double-high attributes. When combined with high-performance Bi_2_O_3_ hexagonal nanotubes, the resulting aqueous battery exhibits an ultrahigh energy density of 115 Wh/kg and an outstanding power density of 9.5 kW/kg among the same kind. Characterizations and simulations reveal the important role of large interlayer spacing intercalation units and mesoporous cages for excellent electrochemical thermodynamics and kinetics. This work represents a milestone in developing “double-high” electrode materials, pointing in the direction for related research and paving the way for their practical application.

## Abstract 2

Mesoporous cubic nanocages, assembled from coupled monolayers with expanded interlayer spacing induced by intercalation, achieve nearly 100% theoretical capacity and robust cycling.

## Introduction

The energy storage technique undoubtedly holds tremendous promise to cope with environmental and energy crises. As a representative energy-storage technique, aqueous batteries have garnered significant attention and witnessed rapid development due to their great potential for large-scale energy storage, fast charge–discharge capabilities, cost-effectiveness, and high safety.^1−7^ Consequently, the electrode materials are the key components determining the battery’s properties. Generally, specific capacity and cycling performance are two crucial indexes in the evaluation of the electrode materials. In terms of specific capacity, the unremitting pursuit of it is to reach 100% of the electrode materials’ theoretical values—in other words, a 100% atom utilization ratio. Unfortunately, that is still far from being achieved so far. As for cycling stability, the current Faradaic-type electrode materials can rarely retain long cycling times.^8,9^ It follows that either the specific capacity or the cycling performance is hardly satisfactory at present, not to mention synchronously achieving their outstanding performance. Therefore, if the approaching theoretical-value capacity and long-term cycling (“double high”) can be simultaneously realized in electrode materials, a new breakthrough will be made for aqueous batteries.

The electrode materials for aqueous batteries include many kinds, and they can be classified into Faradaic-type materials and electrical double-layer type materials according to their energy-storage mechanisms.^10−13^ The former ones, including various transition-metal hydroxides, oxides, and sulfides, have been widely studied due to their much higher theoretical capacitance based on the Faradaic reactions than the latter ones. Among them, Ni(OH)_2_ stands out with the merits of high theoretical capacity (289 mAh/g, 2081 F/g at 0–0.5 V), easy modulation of structures, etc.^14−16^ However, it is challenging for it to approach theoretical capacity or achieve ultrahigh stability, not to mention simultaneously realize both aspects. Thus, great effort has been made to achieve these properties.

The most adopted strategy is scaling down active materials’ sizes, which can improve their specific surface area for electrochemical reactions. As a result, various forms of Ni(OH)_2_, including thin films, nanoparticles, nanorods, and nanosheets, have been prepared and benefitted from the more exposed surface area: the specific capacitances of pure Ni(OH)_2_ materials were effectively boosted to 20–60% of their theoretical values.^17−21^ Nevertheless, that is still far from their theoretical capacitance, which may be because some inner structures and high-surface-energy-induced local agglomerations hinder some atoms from participating in the electrochemical reactions. To overcome that issue, their hierarchical assembled structures, such as nanosheet-assembled flowers, honeycomb-like thin film, nanofiber-assembled hollow spheres, and nanosheet-assembled nanocages, have been acquired.^22−31^ Those exquisite structures further enhance the specific capacitance of pure Ni(OH)_2_ materials, and several can even reach nearly 80% of the theoretical value. It is a pity that their cycling properties are still very poor, generally under a thousand cycling times. Forming composites with highly conductive substrates is another widely used method to concurrently improve the specific capacitance as well as cycling performance, which originates from improved uniformity, higher conductivity, and buffed volume changes. At present, graphene, CNTs, expanded graphite, biomass-derived carbon, carbon nanofibers, carbon fabric, and their composites have been applied as the conductive skeletons to load Ni(OH)_2_ on their surface.^32−39^ Consequently, the majority of their cycling properties are improved to thousands of cycling times aided by those carbon materials, which is still not a satisfactory outcome. More importantly, their specific capacitances are not enhanced fundamentally, which are mostly below 60% of their theoretical values. This limitation arises from the fact that the conductive substrates themselves are often inert, contributing little to the overall capacitance while occupying a certain fraction of the total mass. In addition to the aforementioned strategies, elemental doping, hybridization with other metal compounds, and decoration with active species have also been explored to improve the material properties. However, the specific capacitances achieved by these approaches do not solely originate from Ni(OH)_2_, making them incomparable to pure Ni(OH)_2_ materials. Despite these efforts, none of the Ni(OH)_2_ systems reported to date have been able to achieve both the theoretical capacitance value and ultrahigh cycling stability, whether in pure form or through modifications. The main reason lies in the two aspects being generally contradictory: the high atomic utilization efficiency of electrode materials often requires a high atom exposure ratio, but that will unavoidably cause instability during cycling due to high surface energy. Then can that contradiction be reconciled, and even the limitation be broken? The biomimetic structure design of materials, which mimics the delicate biological structures needed to fulfill particular functions, may provide a possible solution.

To solve the aforementioned puzzle, we adopted the self-template biomimetic method to synthesize brand-new mesoporous Ni(OH)_2_ constructions, which are nearly 100% pure and uniform house-of-cards cubic nanocages assembled by ∼1 nm superlarge *d*-spacing Ni(OH)_2_ coupled monolayers. Such unique mesoporous structures bring about complete atom exposure on the surface with a superhigh specific surface area of 505 m^2^/g, which is the largest and far beyond others among all the reported Ni(OH)_2_ materials, even the metal (hydr-)oxides. Furthermore, it possesses a two-level assembly, coupled monolayer structural units, and house-of-cards nanocages assembled by crisscross structural units. As a result, it delivers an amazing specific capacity approaching nearly 100% of the theoretical value and a remarkable cycling performance of 10,000 times. The underlying reasons for its peerless electrochemical property were revealed through detailed structural characterizations, electrochemical tests, and DFT simulations. This work obtains brand-new Ni(OH)_2_ structures, which not only perfectly reconcile the contradiction between capacity and cycling but also breaks through the ceiling, thus fulfilling the peerless “double-high” performance. The realization of “double high” brings revolutionary changes for the aqueous battery area and possesses important guiding significance for other electrode materials. Due to its outstanding merits, including facile synthesis, mild condition, atomic economy, green technology, and low cost, it also holds great industrial prospects.

## Results and Discussion

### Biomimetic Synthesis Design of NiCMCs

**Figure 1:**
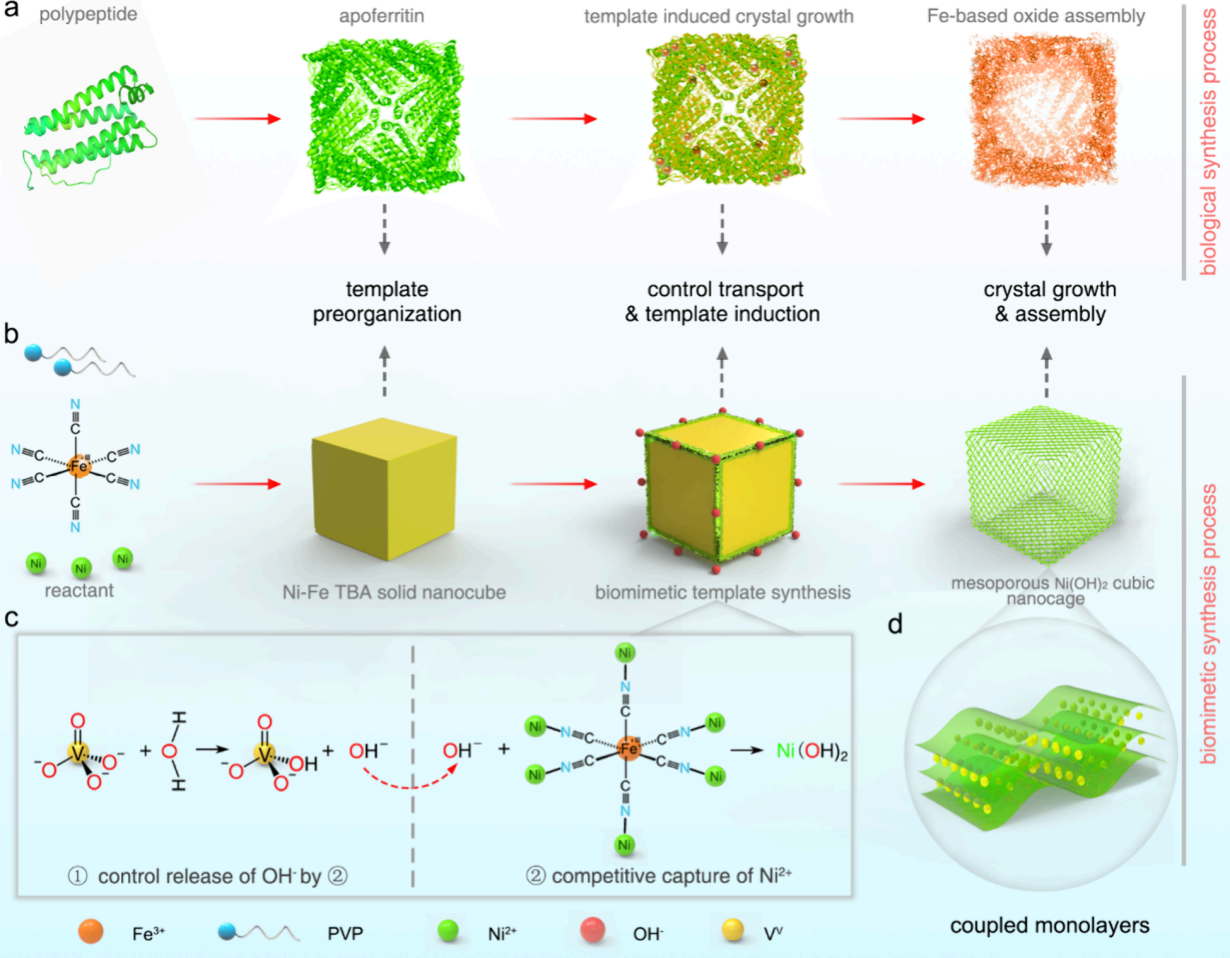
Biomimetic synthesis of NiCMCs. (a, b) Schematic diagrams of biological synthesis process of Fe-based oxide in apoferritin, and corresponding biomimetic synthesis process of NiCMCs. (c) Two-step control transport process during biomimetic synthesis. (d) Schematic diagram of a Ni(OH)_2_ coupled monolayers building block.

To achieve the double-high goal, an exquisite monolayer assembly featuring a nearly 100% atom exposure ratio is necessary. However, the desired structure is not available at all currently. Nature is the best teacher. The ubiquitous Fe storage process in apoferritins, which can produce iron oxide assembled structures with high atomic exposure, provides synthesis inspiration for us to overcome that difficulty.^40^ The Fe storage process in apoferritins involves three steps: (1) the preorganization of polypeptides into cubic-symmetry hollow nanocages composed of 24 protein subunits; (2) controlled transport of iron ions through eight hydrophilic channels of apoferritins, followed by surface adsorption, electron transfer, and molecular assembly nucleation; (3) crystal growth and assembly into highly dispersed cubic-symmetry hierarchical nanostructures under template induction effects (Figure 1a). When mimicking the above biological process to obtain high-atom-exposure Ni(OH)_2_, there are several obstacles to overcome and techniques to build (Figure 1b).

**(1) Screening for suitable preorganized templates.** The selection of templates was challenging because they must meet several criteria simultaneously, including appropriate ion binding strength favoring templates’ assembling and disassembling, the ability to form ion-controlled transport channels, uniform size, good dispersion, and high purity. We discovered that [Fe(CN)_6_]^3–^ had regular octahedral structures that could combine with Ni^2+^ to form Ni–Fe TBA nanocubes using controlled assembly techniques (Figure S1). They are ideal templates due to their suitable ion binding strength and distinct ability to form uniform and well-dispersed full cubes.

**(2) Constructing a Ni(OH)_2_ cubic framework that easily forms biomimetic ion transport channels.** The biggest challenge is to avoid structural collapse of the newly formed Ni(OH)_2_ when removing the templates. We tactfully controlled the speed balance so that the new structures reconstructed as the old templates dissolved. As a result, the above difficulty is resolved perfectly (Figure 1c, d).

**(3) Reassembling hierarchical loose structures with 100% atomic exposure.** To obtain such demanding structures, we adopted high surface exposure biomimetic strategy for both the building blocks and assembly mode. Specifically, the coupled monolayers with extended *d*-spacing were used as building blocks, and all the atoms in the building block allowed to contact the electrolyte ions during the electrochemical reaction benefitted from the ultrathin and spacing-increased structures, making them actually fully exposed. On the other hand, the house-of-cards-like crisscross assembly mode of the building blocks forms loose structures, preventing restacking, generating more mesopores, and providing superior robustness. To sum up, we used the above three steps to successfully realize the biomimetic synthesis of the perfect biological process, which is expected to produce the desired structures.

### Structural Characterizations of NiCMCs

**Figure 2:**
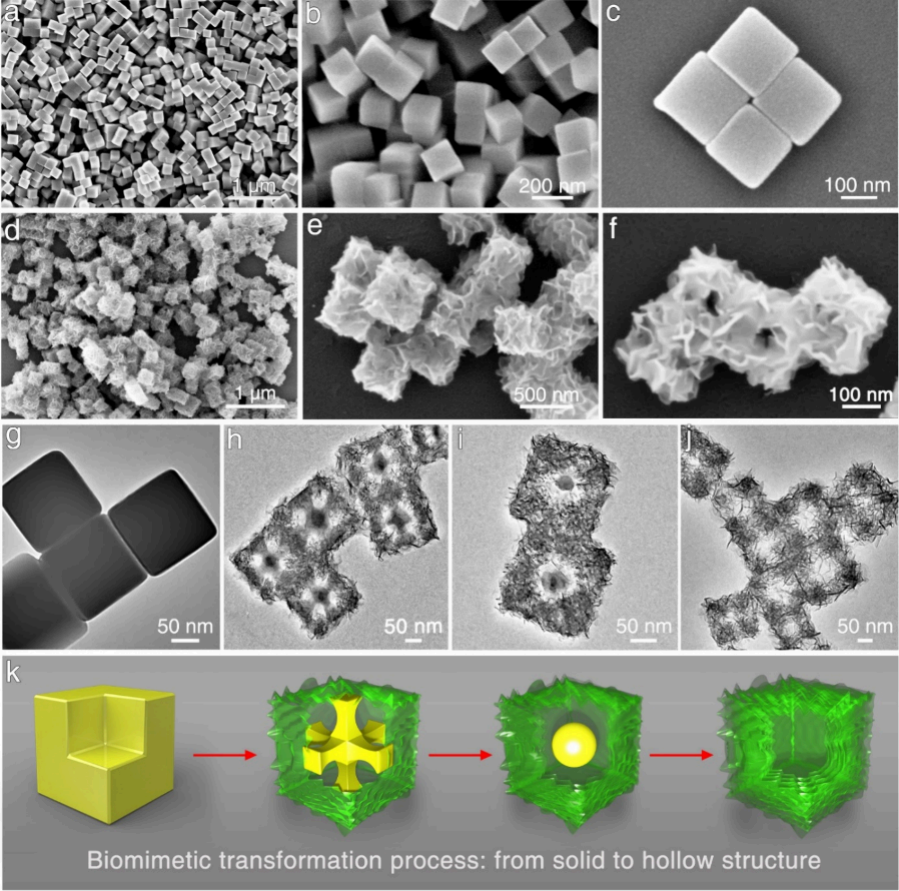
Morphology and structure characterizations of products during biomimetic transformation. (a–c) SEM images of preorganized Ni–Fe TBA nanocube templates. (d–f) SEM images of NiCMCs. (g–k) TEM images of intermediate products during biomimetic structural evolution process and corresponding schematic (yellow represents Ni–Fe TBA, and green represents NiCMC).

**Figure 3:**
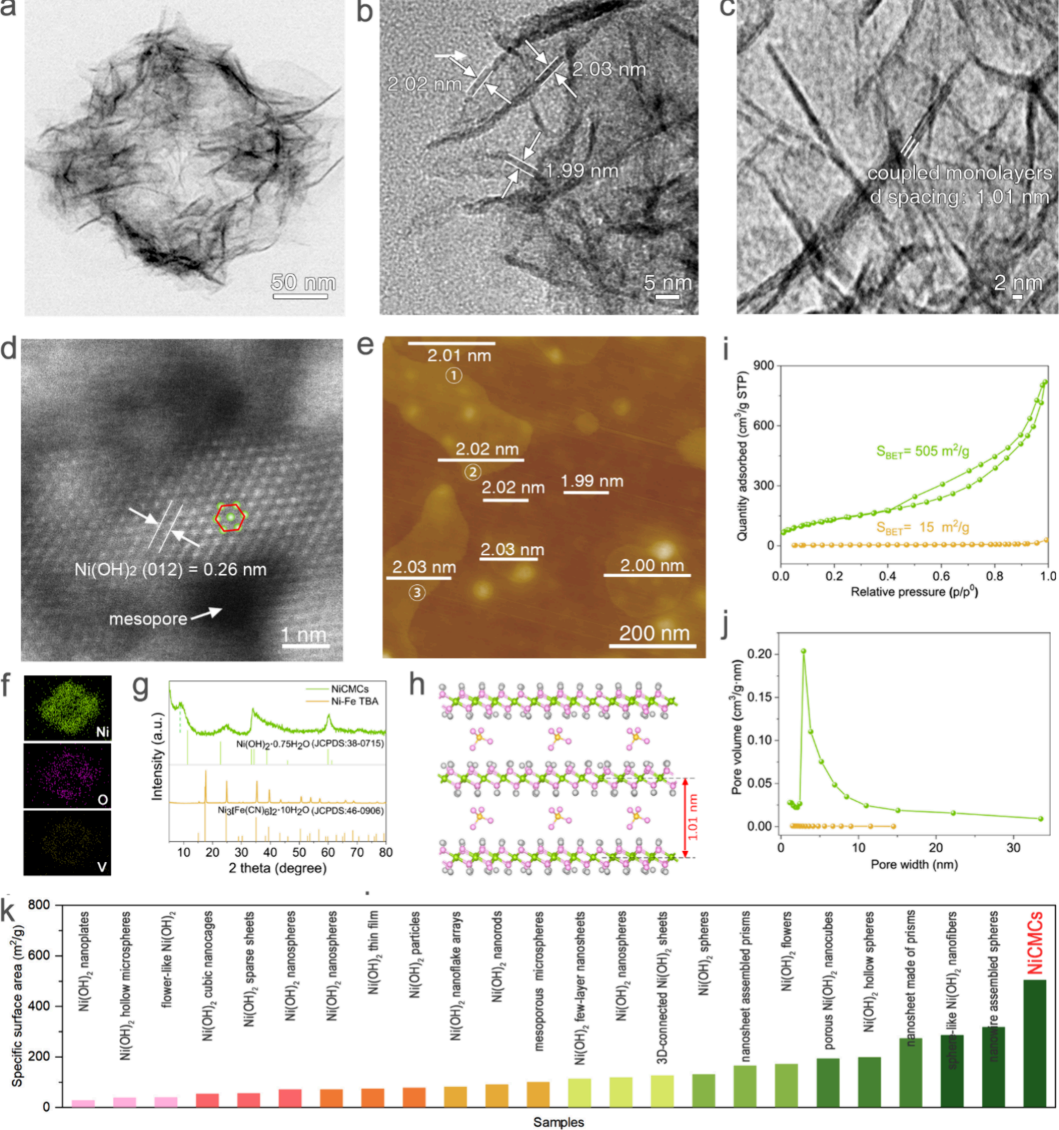
Microstructure characterizations of NiCMCs. (a–c) TEM images showing fine structures (a), thicknesses (b), and coupled monolayers building blocks (c) of NiCMCs. (d) HAADF-STEM image of NiCMCs. (e) AFM image of NiCMCs. (f) Elemental mapping images of NiCMCs. (g) XRD patterns of Ni–Fe TBA and NiCMCs. (h) Schematic of coupled monolayers structure of NiCMCs. (i) Nitrogen adsorption isotherms and (j) pore size distributions of Ni–Fe TBA and NiCMCs. (k) Specific surface area comparisons of various structured Ni(OH)_2_ reported and NiCMCs.^19,22,23,25−31,41−52^

To make clear the formation process of the nanoflake-assembled Ni(OH)_2_ hollow cubes, time-dependent experiments were carried out, and intermediate products were captured and characterized. Figure 2g–k displays the structures of the collected products at different transformation times, along with corresponding schematics representing three typical stages. When the solid Ni–Fe TBA cube templates with smooth surfaces encounter OH^–^ ions generated from the hydrolysis of Na_3_VO_4_ (Formulas 1, 2), the transformation process begins (Figure 2h). Initially, the reaction predominantly occurs at the eight corners and 12 edges of the nanocube templates due to those positions’ high reaction activity resulting from high surface energy (Figures S2, S3). As a result, OH^–^ ions will compete with [Fe(CN)_6_]^3–^ ions, replacing them by combining with Ni^2+^ at the corners and edges of the templates, leading to the in situ formation of Ni(OH)_2_ ultrathin nanoflakes on the templates (Figure 2i, Formula 2). Then, the speed of OH^–^ ion transfer was controlled by the in situ formed Ni(OH)_2_ nanoflakes due to the space-confinement effect, which is exactly the same as our design and speculation. As OH^–^ ions pass through the corners and edges and move toward the body center of the cubes, the constituent of the templates from the edges to the body center is gradually depleted, leaving six pillar structures with a core composed entirely of Ni_3_[Fe(CN)_6_]_2_ (Figures 2h, 2k_2_, S4–S6). These six pillar structures acted as supports, preventing the collapse of the nanocube templates during transformation. Subsequently, OH^–^ ions will further react with the six pillars from the outside to the inside, forming a Ni_3_[Fe(CN)_6_]_2_ sphere interior core (Figure 2i, k_3_). Finally, when the interior core also undergoes the reaction, the transformation was complete, resulting in a light blue product with good dispersibility and hollow structures (Figures 2j, 2k_4_, S7–S10). More importantly, the hollow Ni(OH)_2_ structures are found to be highly loosened. Such loosened structures can not only effectively prevent the agglomeration of building blocks for the highest surface exposure possible but also generate numerous mesopores and gaps for better electrolyte diffusion. Then how do such loosened and hollow structures form? For the loosened structures, its formation results from the large ion volume difference brought by the replacement of [Fe(CN)_6_]^3–^ by OH^–^. In terms of the hollow structures, besides the ion volume difference, the directional ion replacement from the outside (edges and corners) to the inside is the key. The complete formation mechanism and process are illustrated in Figure 2k. It is just because of this biomimetic transformation process that we basically get our target products (Figures S11–13).
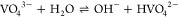
1
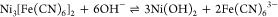
2It is necessary to conduct further research on the fine structure, fraction void, and atomic exposure degree of the nanoflakes within a single house-of-cards cubic nanocage. A high-magnification TEM image reveals the highly transparent nature of a single nanocage. The nanocage is composed of ultrathin flakes assembled in a crisscross pattern like a house of cards, ensuring stability and fixation of the assembly system while preventing flake agglomeration and resulting in a large specific surface area (Figure 3a, b). Measurements at different locations indicate an average thickness of approximately 2 nm for the ultrathin flakes (Figure 3b). A high-resolution TEM (HRTEM) image of the cross sections of the ultrathin flakes shows a clear structure of coupled monolayers with a *d*-spacing of around 1.01 nm (Figure 3c). Notably, this *d*-spacing value is much larger than the interlayer space of 0.78 nm for typical layered Ni(OH)_2_, which may result from the intercalation of VO_4_^3–^ into interlayers. Such a large interlayer space completely allows the free flow of electrolyte ions, which is actually equivalent to the utilization effect of a single layer so that its utilization rate reaches the extreme. The selected-area electron diffraction (SAED) spectrum of the flakes exhibits two round haloes, indicating their polycrystalline and weak crystallization structures (Figure S14). High-angle annular dark-field scanning transmission electron microscopy (HAADF-STEM) imaging was used to observe the atomic-resolution structures of the ultrathin flakes. The image displays a locally ordered atomic structure arranged in a hexagonal pattern, along with some mesoporous structures (defects), which facilitate both high electronic and high ionic conductivity within the ultrathin flakes (Figure 3d).^53^ To further characterize its flakes, atomic force microscopy (AFM) analysis was conducted at multiple locations. The thicknesses of the ultrathin flakes in the wide view were measured to be 2.0 ± 0.1 nm, confirming the coupled structure of monolayers and the high uniformity of the flakes (Figures 3e, S15). This result aligns well with the TEM findings.

Further characterization was conducted to elucidate the composition, microstructure, and valence electron state of the material. EDS analysis is utilized to characterize the element compositions and distributions of the NiCMCs. The EDS image further confirms the abundance of Ni and O, along with a small amount of V (Figure S10). These elements exhibit uniform intensity distributions on the exterior and weaker distributions on the interior, providing additional confirmation of the nanocage structures (Figure 3f). The precursors’ XRD pattern displays sharp diffraction peaks with strong intensity, indicating their high crystallinity (Figure 3g). The peak positions and intensities are in good agreement with those of pure-phase cubic Ni_3_[Fe(CN)_6_]_2_·10H_2_O (JCPDS card, No. 46-0906). In contrast, the NiCMCs’ XRD pattern exhibited only low-intensity broadened diffraction peaks, which aligns with the SAED results. These peaks predominantly correspond to α-Ni(OH)_2_, suggesting partially crystalline structures resulting from the nanosize effect of the ultrathin flake building blocks. The interlayer space is calculated to be 1.01 nm based on the peak observed at 8.7°, consistent with the TEM and AFM findings (Figure 3g). The intermediate products reveal peaks of Ni_3_[Fe(CN)_6_]_2_ and Ni(OH)_2_, indicating that no other substances form during the transformation process (Figure S16). Both the Raman and FT-IR results confirm the presence of Ni(OH)_2_ and VO_4_^3–^ (Figures S17, S18). All of the aforementioned results indicate that NiCMCs are assembled by Ni(OH)_2_ coupled monolayers with VO_4_^3–^ intercalation (Figure 3h). The intercalation of VO_4_^3–^ ions serves to enlarge and stabilize the interlayer spacing. Coupled with the hierarchical pore structures of NiCMCs, it can effectively alleviate volume changes during electrochemical energy storage reactions, potentially enhancing the cycling stability of electrode materials.

The specific surface areas (SSAs) and pore parameters were determined using nitrogen adsorption isotherms (Figure 3i, j). The isothermal curve of the Ni–Fe TBA precursor templates shows minimal N_2_ adsorption, indicating a small SSA and few pores. In contrast, NiCMCs exhibit high N_2_ adsorption, and their isotherm shows a type IV pattern with an H3-shaped hysteresis loop. The type IV isotherm suggests the coexistence of micro- and meso-pores, while the H3-shaped hysteresis loop indicates the presence of slit pores formed by plate-like structures, consistent with the SEM and TEM results. The SSA of Ni–Fe TBA is only 5 m^2^ g^–1^, whereas the SSA of Ni(OH)_2_ is improved by 100 times to an amazing value of 505 m^2^ g^–1^, which approaches its theoretical SSA (see the Experimental Section; Table S1). This value is the largest and far beyond others among all reported Ni(OH)_2_ materials, even the metal (hydr-)oxides (Figure 3k, Table S2). These results, along with the TEM and AFM findings, confirm that nearly 100% of the atoms are exposed to the surface. Such a high SSA can effectively increase the solid–liquid contact interface between the electrode and electrolyte to achieve a high charge transfer rate during the charge storage. process. Furthermore, the pore volume of NiCMCs is also significantly increased by nearly 200 times to 1.1 cm^3^ g^–1^ compared to 0.006 cm^3^ g^–1^ of Ni–Fe TBA templates. More importantly, these pores are distributed within a broad mesoporous range of 2–20 nm (Figure 3k). Those abundant mesopores can concurrently enhance both charge transfer and mass transport-diffusion processes, thereby greatly improving the Faradaic reactions during energy storage.

**Figure 4:**
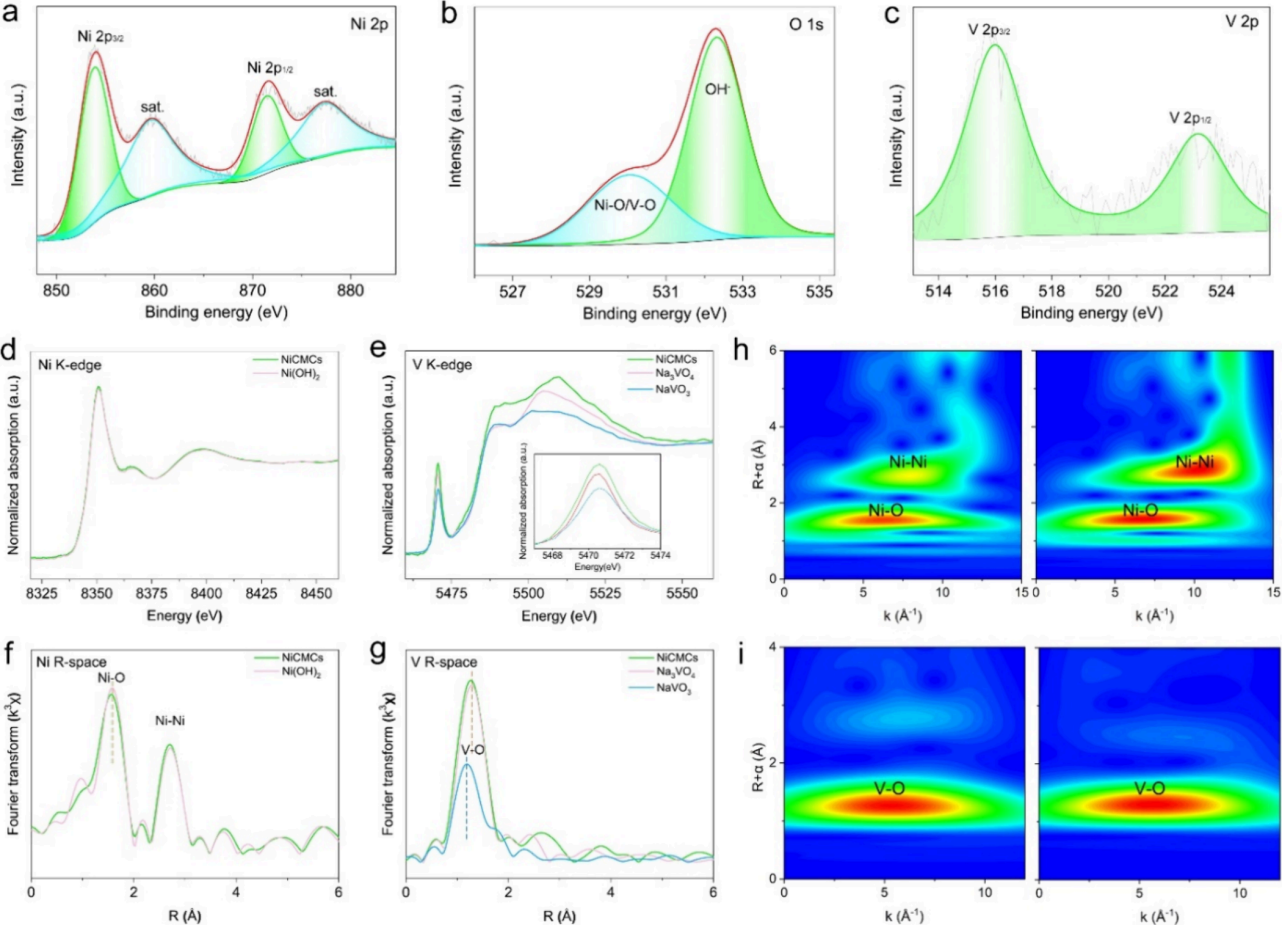
Electronic characterizations of the NiCMCs. (a) Ni 2p, (b) O 1s, and (c) V 2p XPS high-resolution spectra of NiCMCs. (d) Ni and (e) V K-edge XANES spectra. (f, g) Fourier-transformed (f) Ni K-edge and (g) V K-edge EXAFS spectra. (h) WT-EXAFS of Ni of the as-prepared NiCMCs, and commercial Ni(OH)_2_ (from left to right). (i) WT-EXAFS of V of the as-prepared NiCMCs, and commercial Na_3_VO_4_.

The surface chemical states of the elements in the NiCMCs were investigated further by using XPS. The results confirm the presence of Ni, O, and V elements in the NiCMCs (Figure 4a–c), consistent with the EDS analysis. The high-resolution Ni 2p spectrum reveals Ni 2p_3/2_ and Ni 2p_1/2_ peaks with two shakeup satellites, located at 854.0 and 871.6 eV, respectively (Figure 4a). The energy separation of 17.6 eV between the Ni 2p_3/2_ and Ni 2p_1/2_ peaks is a typical characteristic of Ni(OH)_2_.^54^ The O 1s peak can be divided into two peaks at 532.4 and 530.0 eV, associated with absorbed OH^–^ and lattice O in Ni(OH)_2_, respectively (Figure 4b). The V 2p profile can be deconvoluted into V 2p_3/2_ and V 2p_1/2_ peaks, with binding energies of 516.0 and 523.3 eV, respectively, consistent with V in VO_4_^3–^ (Figure 4c).^55^ These findings are in agreement with the aforementioned analysis, confirming the formation of VO_4_^3–^-intercalated Ni(OH)_2_.

To elucidate the local atomic coordination and electronic structure of NiCMCs, we conducted X-ray absorption near edge spectroscopy (XANES) measurements. As depicted in Figure 4d and 4e, the Ni K-edge spectra of the NiCMCs and commercial Ni(OH)_2_ exhibit remarkable similarity, indicating comparable Ni valence states.^56^ However, notable distinctions can be observed in the V K-edge spectra of NiCMCs compared to commercial Na_3_VO_4_ and NaVO_3_, suggesting an influence of V intercalation on its coordination. Specifically, the V K-edge XANES spectrum of NiCMCs exhibits heightened pre-edge peaks (inset in Figure 4e), indicating a more distorted coordination environment around the V atoms in NiCMCs compared to commercial Na_3_VO_4_ and NaVO_3_. The corresponding Fourier-transformed extended X-ray absorption fine structure (FT-EXAFS) spectra provide further insights into bonding lengths and coordination states. For the Ni K-edge FT-EXAFS of both NiCMCs and commercial Ni(OH)_2_, two prominent coordination peaks with similar intensity are observed at ∼1.5 and ∼2.7 Å, attributed to the Ni–O peak and Ni–Ni peak (Figure 4f).^57^ However, the two peaks of NiCMCs slightly shift toward a shorter distance compared to those of commercial Ni(OH)_2_. This observation suggests structural contraction in the ultrathin nanosheets of NiCMCs, and the surface distortion of these nanoflakes helps to balance their excessive surface energy, ultimately contributing to their excellent stability. Regarding the FT-EXAFS curves of the V K-edge, all of the samples exhibit a prominent V–O peak (Figure 4g). However, the NiCMCs and Na_3_VO_4_ display similar bond length and peak intensity, which are both larger than those of NaVO_3_. Similar trends are observed in the Wavelet transform EXAFS (WT-EXAFS) analysis of the Ni K-edge and V K-edge data (Figure 4h, i). Notably, the EXAFS WT maps of Ni of NiCMCs and commercial Ni(OH)_2_ show obvious peak intensity differences at ∼2.5–3.0 Å, which could be attributed to their construction difference. For the EXAFS WT maps of V of NiCMCs and commercial Na_3_VO_4_, their peak intensities and positions are very similar, indicating the V is intercalated in the interlayer rather than doped into the crystal lattice.^58^ These findings collectively confirm the VO_4_^3–^ inserted Ni(OH)_2_ coupled monolayer structures in NiCMCs.

All of the aforementioned characterizations have demonstrated the successful preparation of ideal Ni(OH)_2_ structures featuring complete uniformity. Such exquisite structures are achieved through an ingenious biomimetic synthesis method, which lays a good foundation for peerless double-high properties.

### Electrochemical Performance

**Figure 5:**
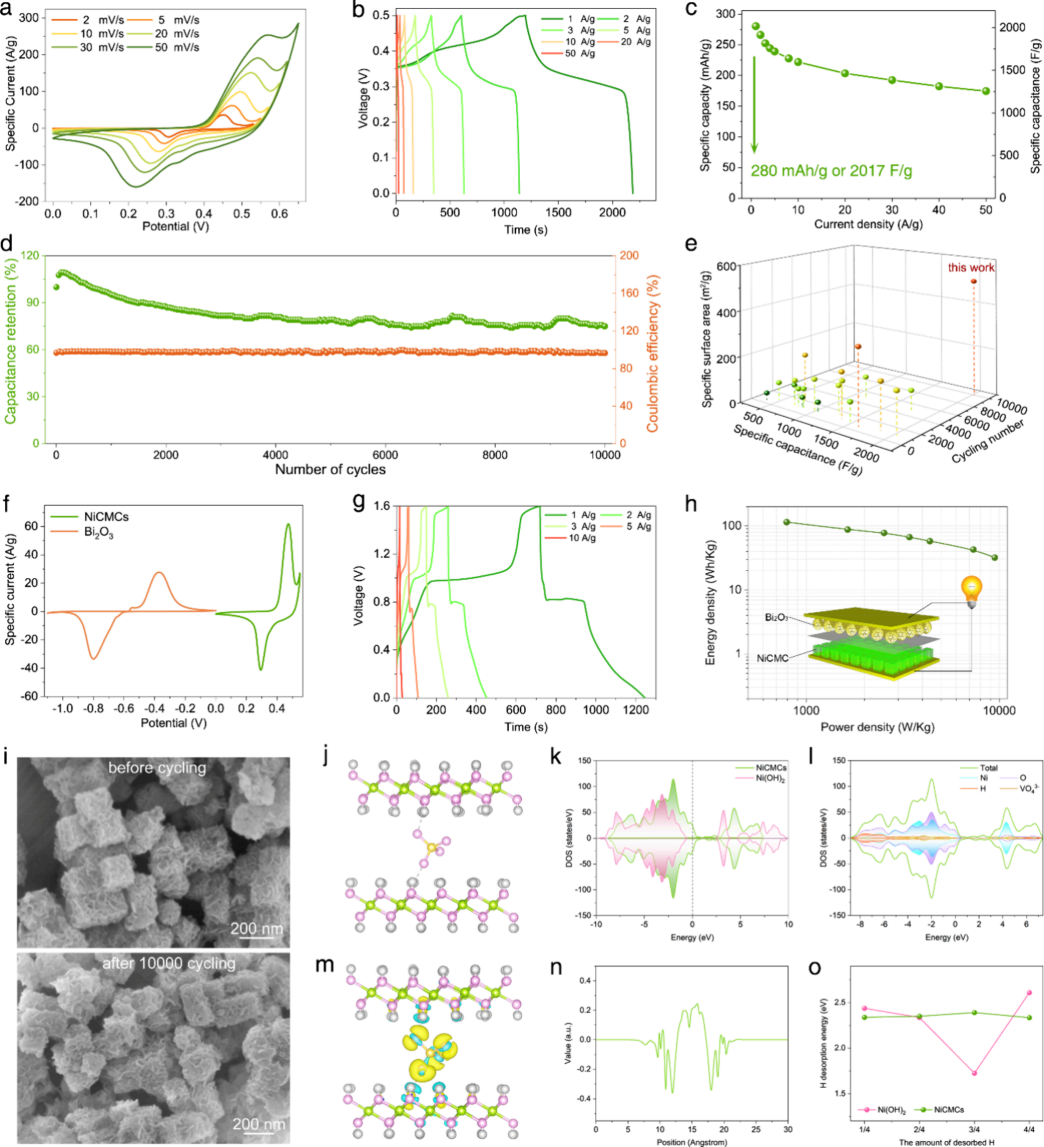
Electrochemical properties and mechanisms of NiCMCs. (a) CV curves at different scan rates. (b) GCD curves at various current densities. (c) Specific capacities at different current densities. (d) Cycling stability after 10,000 cycles. (e) Comprehensive comparison of various reported Ni(OH)_2_-based electrode materials and NiCMCs. (f) CV curves of NiCMCs and Bi_2_O_3_. (g, h) GCD curves (g) and Ragone plot (h) of NiCMCs//Bi_2_O_3_ hybrid devices. (i) SEM images of NiCMCs before and after 10,000 charge–discharge cycles. (j) The optimized atomic structure model of NiCMCs. (k) The density of states (DOS) of NiCMCs and Ni(OH)_2_. (l) The local DOS of NiCMCs. (m) Charge density difference of NiCMCs, where the yellow and blue represent electron accumulation and depletion, respectively. (n) The plane-averaged charge density difference along the Z direction of NiCMCs. (o) HDEs as a function of the amount of desorbed H for NiCMCs and Ni(OH)_2_.

To verify whether the above nanocages with a highly exposed surface can achieve double-high properties, electrochemical studies were conducted. The specific capacity, which is a key index for evaluating electrode electrochemical properties, was measured by using NiCMCs as cathode materials in a 6 M KOH solution with a three-electrode system. The NiCMCs exhibit a wide and stable potential window from 0 to 0.6 V, as shown in the cyclic voltammetry (CV) curves (Figure 5a). Meanwhile, a pair of redox peaks appears in all the CV curves, manifesting their Faradaic-type electrochemical behavior. The pair of redox peaks correspond to the conversion reaction between Ni^2+^ and Ni^3+^: Ni(OH)_2_ + OH^–^ ⇌ NiOOH + H_2_O + *e*^–^. The similar shape of the CV curves with increased scan rates suggests an excellent rate capability. The NiCMCs’ specific capacitance and rate capability were further quantified using the galvanostatic charge–discharge (GCD) method. GCD curves were measured at current densities ranging from 1 A g^–1^ to a high value of 50 A g^–1^ (Figure 5b). Each GCD curve displays a pair of potential plateaus, further confirming their Faradaic-type energy storage mechanism and aligning well with the redox peaks observed in the CV curves in terms of potential positions. Notably, the GCD curves exhibit high symmetry, indicating reversible redox reactions without side reactions. Meanwhile, the potential plateaus in the GCD curves are not so flat with a certain slope, which may be due to the surface capacitive charge storage brought by coupled monolayer assembled structures. The specific capacities are calculated from the GCD curves (Figure 5c). An impressive specific capacity value of 280 mAh/g is obtained at a current density of 1 A g^–1^, far surpassing all the reported Ni(OH)_2_ materials (Tables S2, S3). The capacity value mainly stems from the Faradaic reaction, as its mesoporous structure is dominant, rendering the contribution of the electrochemical double-layer capacitance almost negligible. Based on further tests and calculations (Table S4), the capacity of existing Ni(OH)_2_ component in NiCMCs reached 99.7% of its theoretical value, almost approaching the limit of atomic utilization. Even at a current density increased by 50 times to 50 A g^–1^, the NiCMCs retain a specific capacity of 161 mAh/g, which is still a super high value (Figure 5c). Therefore, the near 100% theoretical capacity and 100% atomic utilization are verified mutually.

Cycling stability, another crucial metric for batteries, was evaluated by conducting continuous GCD tests at a high current density of 10 A g^–1^. Impressively, based on the usual cyclic evaluation criteria, the NiCMCs performed over 10,000 cycles, indicating excellent electrochemical stability that significantly surpasses various reported Ni(OH)_2_ electrode materials (Figure 5d). These other materials struggle to achieve good stability even at the cost of sacrificing capacity, not to mention those with relatively high capacity (Figures 5e, S19; Tables S2, S3). So, what fundamentally changes the characteristics of metal oxides that are typically difficult to maintain long-term cycling? Based on the aforementioned analysis of material assembly, the exceptional cycling performance can be attributed to the two-level stability structure. The coupled monolayers serve as the first-level self-stability structures by assembling two monolayers back to back, effectively reducing surface energy while ensuring a high atomic exposure ratio. The cubic nanocages, on the other hand, act as the second-level stability structures through the house-of-cards crisscross assembly of the coupled monolayers, buffering volume expansion and preventing structural collapse.

The intricate biomimetic structure’s influence on the electrochemical reaction kinetics has been studied using various techniques, including electrochemical impedance spectroscopy (EIS) and normalized capacitive capacitance ratios (Figure S20). The EIS curve reveals a negligible equivalent series resistance (*R*_s_) of only 0.7 Ω as well as a small charge-transfer resistance (*R*_ct_) of 1.3 Ω. Such low resistance for electrode materials significantly enhances conductivity and facilitates fast charge transfer at the electrode/electrolyte interface, thereby endowing NiCMCs with outstanding rate capability, which aligns with their unique structures. Using Dunn’s method, the capacitive contributions to the capacity were further calculated (Figure S21).^59,60^ The green region in Figure S21 represents the capacitive current fraction at 1 mV s^–1^, which contributes 88% of the total charge storage. This result demonstrates that the majority of the capacity originates from the surface capacitive reaction, which concurs with NiCMCs’ coupled monolayer assembled structures. In fact, the surface contribution ratio is quite prominent, with the surface capacity contribution of the vast majority of reported Ni(OH)_2_-based electrode materials being below 80% (Figure S22). So it can be inferred that the biomimetic structures of NiCMCs effectively promote the kinetics of the electrochemical reaction, laying the foundation for achieving double-high performance.

Subsequently, we assembled a hybrid energy storage device using NiCMCs as the cathode and Bi_2_O_3_ as the anode (Figures 5f, S23, and S24). Compared with porous carbon anodes, Bi_2_O_3_ anode materials exhibit a wider voltage window and exceptionally high specific capacity. The hybrid device using a Bi_2_O_3_ anode showed a high voltage of up to 1.6 V and showcased an exceptional rate capability of 10 A/g (Figures 5g, S25). As a result, this device delivered an energy density as high as 115 Wh/kg and a power density up to 9500 W/kg based on the total mass of both the anode and the cathode (Figure 5h). Excellent energy storage performance enabled it to easily power an LED array and a digital electronic clock (Figure S24). Its comprehensive metrics far surpass the hybrid device using carbon anode materials (Figures S26, S27), and even various reported Ni(OH)_2_-based aqueous energy storage devices. Thus, the “double-high” electrode materials can fully demonstrate their dual high characteristics in energy storage devices, laying the foundation for advancing the practicality of energy storage devices.

### Mechanisms of Double-High Properties

As evident from the above results, we have successfully achieved double-high properties, namely, ∼100% theoretical capacity and 10,000 times ultrahigh cycling stability. The reason behind achieving the breakthrough necessarily lies in the special microstructures of the electrode materials.

The mystery to achieving nearly 100% theoretical capacity lies in its completely loosened assembly and coupled monolayers with a large interlayer spacing. Specifically, its intersecting and loosely packed assembly mode effectively prevents the agglomeration of ultrathin assembly units, realizing a specific surface area close to the theoretical value. Moreover, it features large interlayer spacing in its coupled monolayer units, allowing electrolyte ions to pass through them freely. Such structures ensure nearly 100% atomic utilization.

The mystery to its high stability lies in its pronounced chemical reaction’s reversibility and structural stability. From an electrochemical reaction principle standpoint, NiCMCs store and release energy through a single-electron-transfer process, which is essentially reversible. This aspect is evident from the symmetric CV and GCD curves as well as from the Coulombic efficiency curve (Figure 5d). In terms of the structural changes during the electrochemical reaction, whether in a single charge–discharge cycle or after 10,000 cycles, the products consistently maintain their highly uniform house-of-cards cubic cage structures and loosened assembly of ultrathin nanosheets (Figures 5i, S28). In addition, the Raman and XPS characterization results after long cycles also confirm the good preservation and reversibility of the electrode material’s microstructure during the energy storage process (Figures S17, S29). Thus, the reversibility of the chemical reactions, the high uniformity of NiCMCs, and the structural stability during the electrochemical reactions ensure long cycling performance.

The aforementioned mechanism is further validated theoretically through Density Functional Theory (DFT) simulations. Figure 5j displays the optimized atomic structure models of the NiCMCs. The electronic properties were analyzed using density of states (DOS) in the DFT calculations. In comparison to the standard α-Ni(OH)_2_, NiCMC exhibits a higher total DOS near the Fermi level and a smaller bandgap, indicating improved electrical conductivity (Figure 5k). Furthermore, the local DOS of Ni and O atoms in NiCMCs shows an increase near the Fermi level in the presence of VO_4_^3–^, suggesting a positive interaction between intercalated VO_4_^3–^ and the host layers (Figure 5l). This interaction decreases the system’s energy, resulting in a relatively stable bonding state, which aligns with the experimental findings.

Simulations were also performed to examine the electron–electron coupling between VO_4_^3–^ and the Ni(OH)_2_ host layers. The charge density difference (Figure 5m) reveals a notable charge migration at the interface between the VO_4_^3–^ and host layers. Additionally, the plane-averaged charge density difference along the Z direction (Figure 5n) demonstrates that electrons at the interface deviate partially from Ni(OH)_2_, generating an interfacial electric field that effectively shields the electrostatic interactions between OH^–^ and the host layer.^61^ Consequently, NiCMC exhibits exceptional interlayer diffusion kinetics of OH^–^, which contributes to its enhanced electrochemical performance. Moreover, electron depletion and accumulation occur around the Ni and O atoms in the host layer interacting with VO_4_^3–^, thereby strengthening the Ni–O bond and stabilizing the two-dimensional crystal structure.

To comprehend the thermodynamics of the electrochemical reaction in NiCMCs and Ni(OH)_2_, hydrogen desorption energies (HDEs) were calculated. Remarkably, the HDEs of NiCMCs exhibit a smoother variation compared to that of Ni(OH)_2_ (Figure 5o). Furthermore, all of the HDEs of NiCMCs are lower than that of Ni(OH)_2_ at the initial and final hydrogen desorption steps. Consequently, the majority of H detachment in NiCMCs is thermodynamically more favorable than that in Ni(OH)_2_. Thus, the simulation results clearly indicate that the coupled monolayers with larger interlayer spacing enable the double-high electrochemical properties.

## Conclusion

In this study, a brand-new mesoporous NiCMC structure was achieved through biomimetic technology, featuring an unprecedented amalgamation of characteristics: record-breaking capacity approaching 100% of the theoretical value, exceptional cycling stability over 10,000 cycles, complete uniformity, a state-of-the-art specific surface area, larger interlayer spacing, coupled monolayer structural units, and loosened house-of-cards assembly. Such a structure effectively breaks the longstanding trade-off between capacity and cycle stability in electrode materials, marking the first instance of achieving double-high performance in aqueous batteries. Characterization results indicate that its exceptional performance originates from near-limit atomic utilization and structural stability during electrochemical reactions facilitated by the completely uniform biomimetic architectures. These findings serve as an invaluable reference for the synthesis of more electrode materials of this kind. Furthermore, the biomimetic synthesis of the NiCMCs material is mild, highly efficient, and eco-friendly, thus holding promise for large-scale production. In view of the double-high features of NiCMCs and the safety attributes of the aqueous electrolyte employed, it brings hope for supplementing or even replacing current ion batteries with poor safety.

## Methods

### Synthesis of Ni–Fe Turnbull Blue Analogue (TBA) Solid Nanocubes

In a typical synthesis, 1.5 g of polyvinylpyrrolidone (PVP), 0.751 g of nickel acetate tetrahydrate, and 1.25 g of sodium citrate were dissolved successively in 100 mL of deionized water to form solution A. Separately, 0.332 g portion of potassium ferricyanide (III) was dissolved in 100 mL of deionized water to form solution B. Solution B was then added to solution A dropwise under magnetic stirring. The resulting mixed solution was aged for 48 h at room temperature away from light. The product was collected by centrifugation, washed with water, and dried at 70 °C in an oven overnight.

### Synthesis of Mesoporous Ni(OH)_2_ Cubic Nanocages Assembled by Coupled Monolayers

In a typical synthesis, 200 mg of Ni–Fe TBA was directly added to a 100 mL aqueous solution containing 2 g of Na_3_VO_4_. Subsequently, the solution was stirred for 10 min before being placed in a 60 °C oven overnight for reaction. The resulting product was collected by centrifugation, washed with water, and dried at 70 °C in an oven overnight.

### Materials Characterization

The morphologies and microstructures of the prepared samples were observed by using field emission scanning electron microscopy (FE-SEM, Hitachi S-4800) and transmission electron microscopy (TEM, JEOL JEM-2100). Atomic structures were observed using an aberration-corrected STEM equipment (FEI Themis Z). The thicknesses of the ultrathin flakes used for assembling the hollow nanocubes were measured by using atomic force microscopy (AFM, Veeco DI Nanoscope MultiMode V system). The compositions of the samples were obtained by using an energy-dispersive X-ray spectroscope (EDX) attached to the FE-SEM instrument. Elemental mapping images were recorded using an EDX spectroscope attached to TEM. The chemical elements and their binding states were analyzed using an X-ray photoelectron spectroscope (XPS, Kratos AXIS ULTRA DLD). The crystal phases of the samples were analyzed by X-ray diffraction (XRD) using an X-ray diffractometer (Bruker D8 Focus) with Cu Kα radiation (λ = 0.15418 nm) at a voltage of 40 kV and a current of 40 mA. Raman spectroscopy spectra were determined by using an Invia confocal Raman system at a laser wavelength of 514 nm. FT-IR spectra were obtained by using an FT-IR spectrometer (Bruker, VERTEX 70). The XAS data were collected using a Table XAFS-500 (Specreation Instruments Co., Ltd.). Nitrogen adsorption isotherms were collected using a TriStar 3020 at 77 K. The specific surface areas were calculated using the Brunauer–Emmet–Teller (BET) method, and pore size distribution plots were obtained using the Barrett–Joyner–Halenda (BJH) method based on the desorption branch. The theoretical specific surface area (SSA_theory_) of NiCMCs was calculated using a model obtained through DFT simulation, which was simplified to a planar structure for the purpose of calculating the theoretical specific surface area. The calculation formula used was SSA_theory_ = *S*_model_/*m*_model_, where *S*_model_ represents the total surface area of the model and *m*_model_ represents the mass of all atoms in the model. The calculated theoretical specific surface area of the NiCMCs was 532 m^2^/g.

### Density Functional Theory (DFT) Calculation

To verify the effect of interlayer regulation on the electrochemical properties of NiCMCs, the first-principle simulations were carried out using the Vienna ab Initio simulation package (VASP, version 6.3.2).^62^ The Perdew–Burke–Ernzerhof (PBE) exchange–correlation functional and the projector augmented-wave (PAW) pseudopotential were applied to spin-unrestricted geometry optimizations. Partial occupancies of the Kohn–Sham orbitals were allowed using the Gaussian smearing method with a width of 0.2 eV. The electronic energy was considered self-consistent when the energy change was smaller than 10^–6^ eV. A geometry optimization was considered to be convergent when the force change was smaller than 0.03 eV/Å. Grimme’s DFT-D3 methodology with Becke-Johnson damping function was used to describe the dispersion interactions. The model of NiCMCs consists of 32 Ni(OH)_2_ and one VO_4_^3–^. The VO_4_^3–^ inserts into the interlayers of nickel hydroxide in an ionic state, and Na^+^ ions are used to balance charges. The sampling of the Brillouin zone is with only the Γ-point in consideration of the dimension of the supercell, i.e., 1.077 × 1.244 × 3.000 nm^3^.

### Electrochemical Test

A three-electrode system was used to measure the electrochemical properties of the NiCMCs in a 6 M KOH aqueous electrolyte at room temperature by using a CHI660E electrochemical workstation (Shanghai Chenhua Instruments Co.). The working electrode was prepared as follows: 80 wt % of the sample, 10 wt % of acetylene black, and 10 wt % of PTFE were mixed to form a viscous slurry, which was then pressed onto a nickel foam current collector at 10 MPa. The prepared electrodes were dried overnight at 80 °C in a vacuum oven. The sample loaded on the current collector had a geometric surface area of about 1 cm^2^. A platinum foil and a Ag/AgCl electrode were used as the counter and reference electrodes, respectively. Cyclic performance was evaluated using the GCD method at a current density of 8 A/g. In the three-electrode system, the specific capacity was calculated by the formula of *C* = *I*Δ*t*/(3.6 × *m*) using the discharge part of the galvanostatic charge–discharge curve (GCD), where *C* is the specific capacity (mAh/g), *I* is the constant current (A), *m* is the mass (g) of the electrode material, and Δ*t* is the discharge time (s) during the discharge process.

### Assembly and Evaluation of Hybrid Energy-Storage Devices

The devices utilized the NiCMCs material and porous carbon material pressed onto nickel foams as the positive and negative electrodes, respectively. The NiCMCs and Bi_2_O_3_ hexagonal nanotubes^8^ were assembled together and separated by a filter paper separator to form hybrid energy-storage devices. The mass ratio of the positive and negative electrodes was determined using the equation: *m*_+_/*m*_–_ = (*C*_–_ × Δ*E*_–_)/(*C*_+_ × Δ*E*_+_). For comparison, porous carbon materials were used as anode materials to assemble hybrid energy-storage devices with NiCMCs. The electrochemical measurements of the devices were carried out in a two-electrode cell at room temperature in a 6 M KOH aqueous electrolyte solution.

## Supporting Information Available

Crystal structure of Ni–Fe TBA; XRD, SEM, EDS, and TEM images of intermediate products; photographs of Ni–Fe TBA solid nanocube solution and NiCMCs solution; zeta potential, Raman image, and FT-IR spectrum of NiCMCs; comparison of electrochemical properties of various samples (PDF)
