# Supplementary material for: Mesoporous Cubic Nanocages Assembled by Coupled Monolayers With 100% Theoretical Capacity and Robust Cycling
Source: ACS Cent Sci. 2024 Jun 10;10(6):1283–94. doi: 10.1021/acscentsci.4c00345 (PMC11212129; doi:10.1021/acscentsci.4c00345)
Supplement: Supplementary file 1 — oc4c00345_si_001.pdf [file oc4c00345_si_001.pdf]

Supporting Information

Mesoporous Cubic Nanocages Assembled by Coupled Monolayers With 100% Theoretical Capacity and Robust Cycling

Guangtao Zan, [†,‡] Shanqing Li, [¶] Ping Chen, [§] Kangze Dong,[†] Qingsheng Wu[†], and Tong Wu[*,†]

[†] School of Chemical Science and Engineering; Institute of Advanced Study; Shanghai Key Laboratory of Chemical Assessment and Sustainability; Tongji University, Shanghai 200092, PR China

[‡] Department of Materials Science and Engineering, Yonsei University, Seoul 03722, Republic of Korea

[¶] School of Materials and Environmental Engineering, Chizhou University, Chizhou, Anhui 247000, PR China

[§] School of Chemistry and Chemical Engineering, Anhui University, Hefei, Anhui 230601, PR China

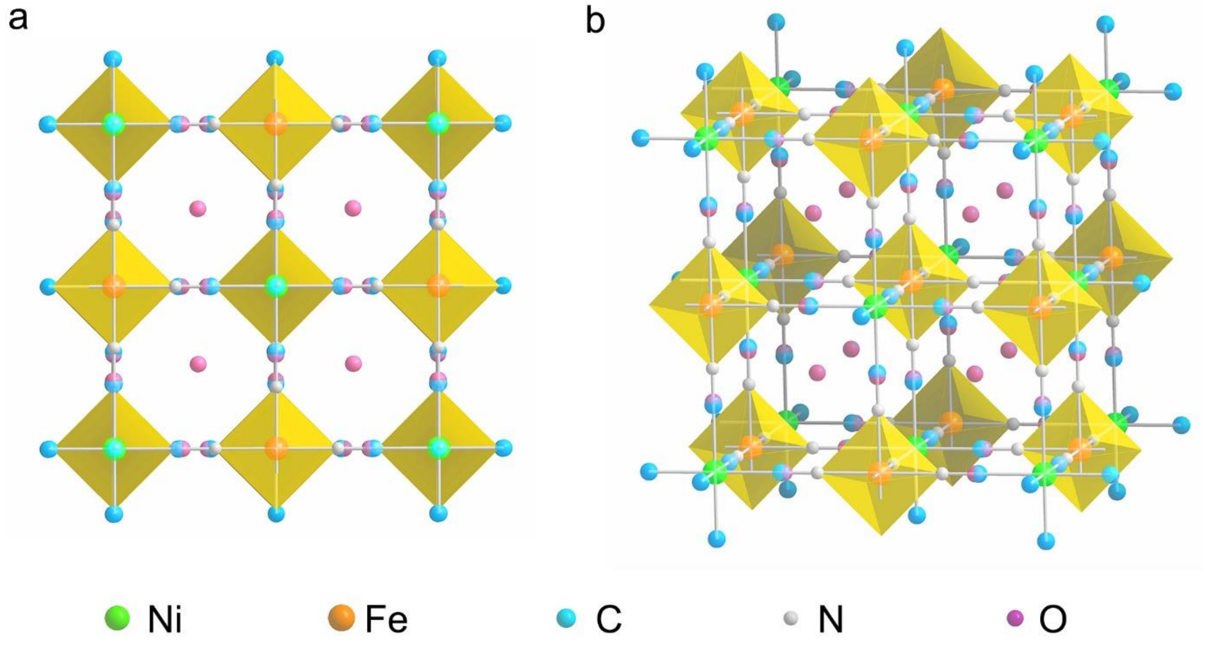

Fig. S1 Crystal structure of Ni-Fe TBA from different views

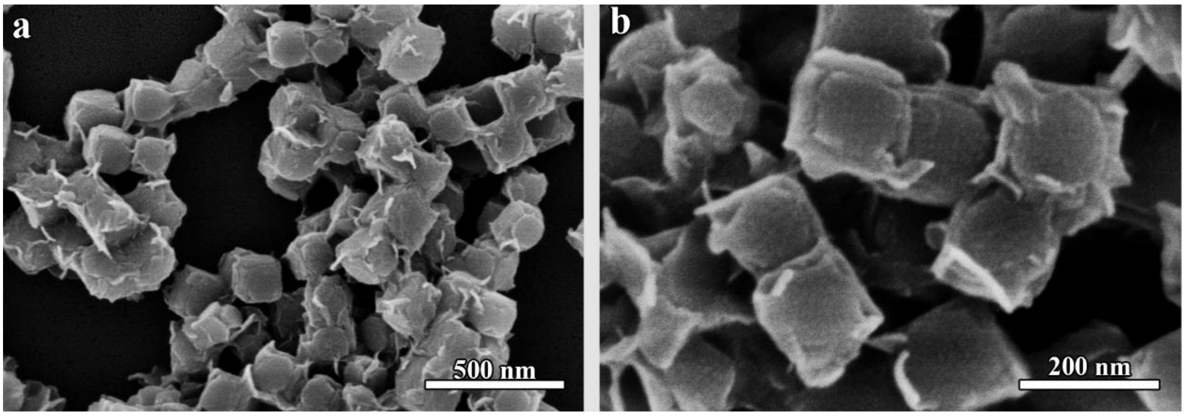

Fig. S2 SEM images of intermediate products at initial stage of the biomimetic transformation process at different magnifications.

Results show that the angles and edges of the Ni-Fe TBA solid nanocubes are first etched and the ultrathin nanoflakes are formed on the surface of the nanocube, consistent with the TEM results.

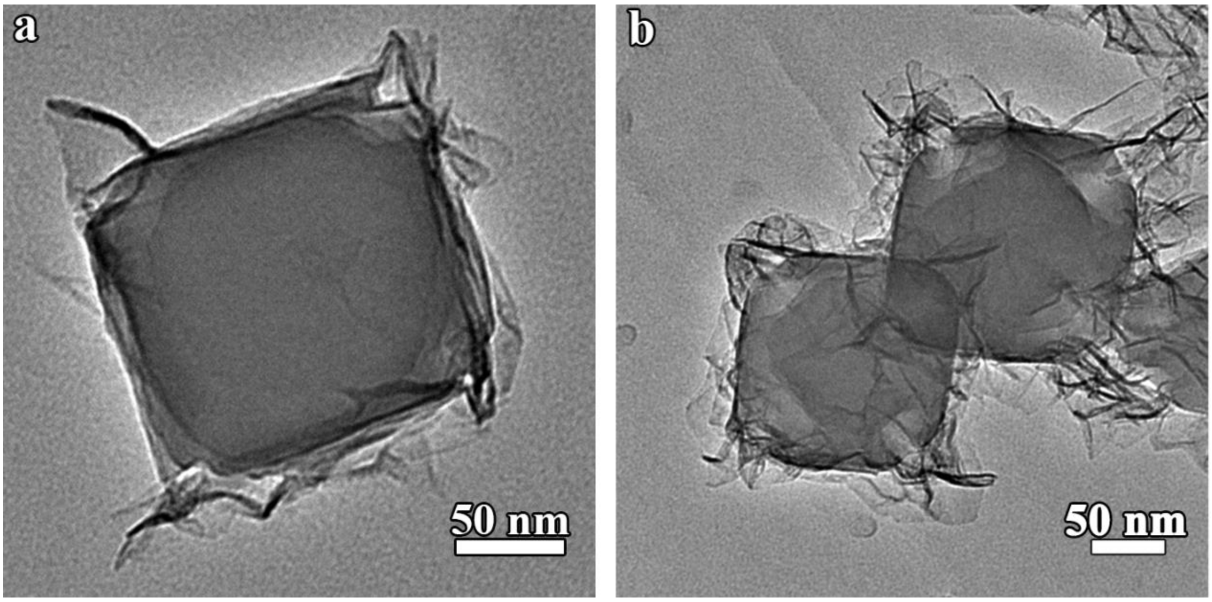

Fig. S3 TEM images of products at initial stage of the biomimetic transformation process. (a) The reaction starts from the angles and edges of the Ni-Fe TBA solid nanocube. (b) The Ni-Fe TBA solid nanocube is further etched from the angles and edges to the core.

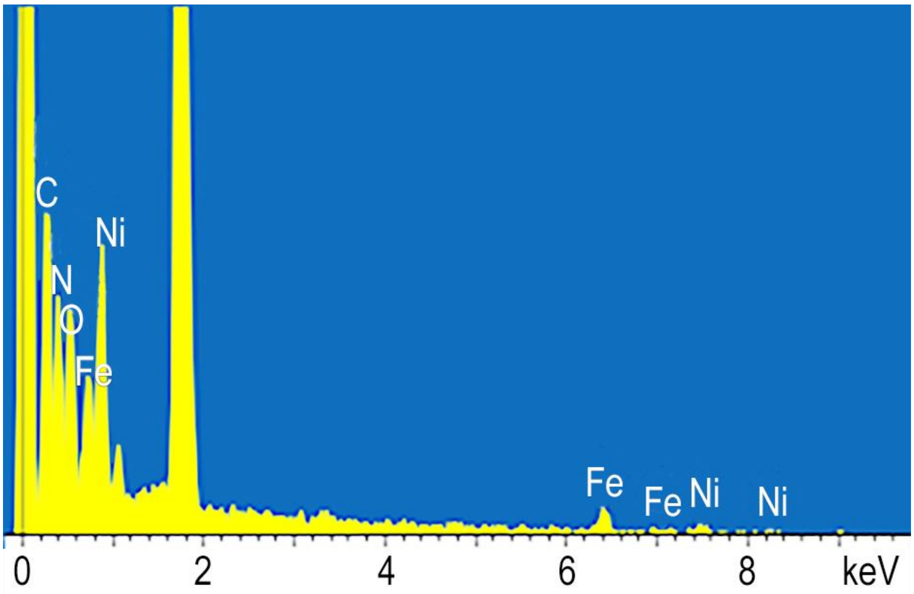

Fig. S4 EDS image of Ni-Fe TBA solid nanocubes

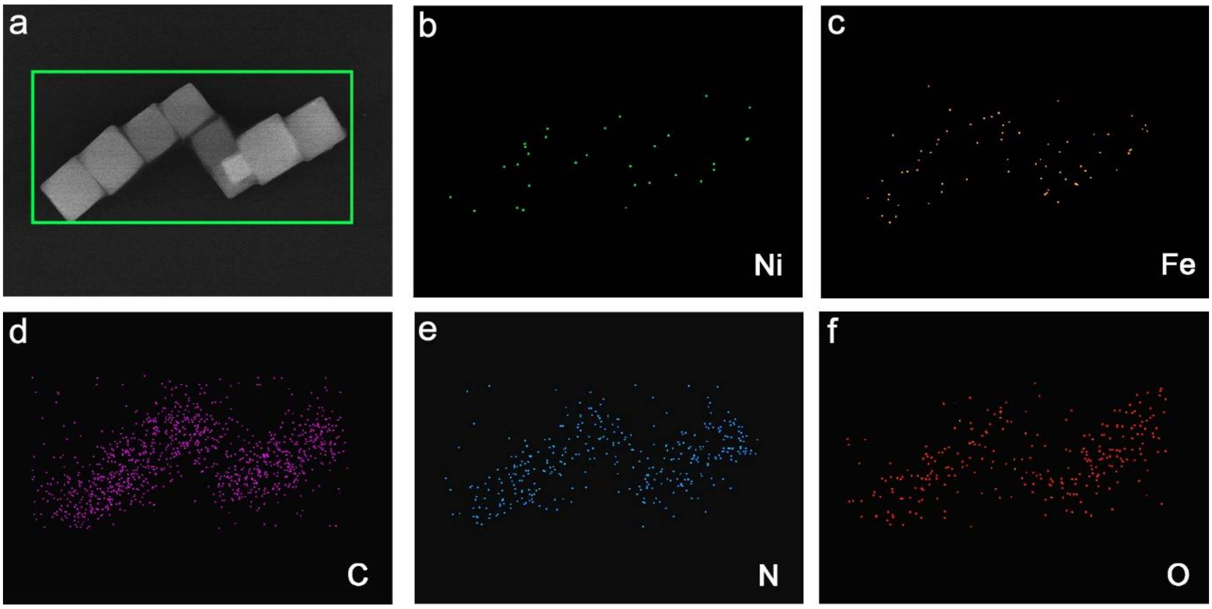

Fig. S5 Elemental mapping of Ni-Fe TBA solid nanocubes.

Results indicate the uniform distributions of all the elements.

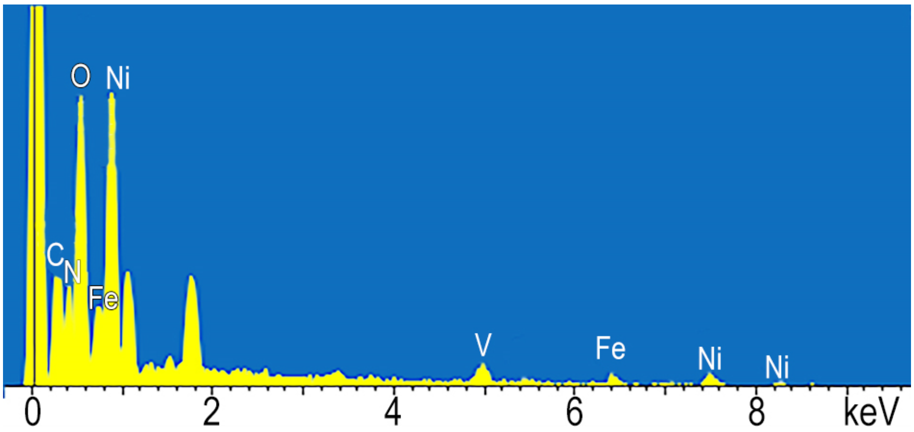

Fig S6 EDS pattern of intermediate products during biomimetic transformation of Ni-Fe TBA to form NiCMCs.

Compared to Ni-Fe TBA, the EDS spectrum of the intermediate products exhibits an increased Ni content and decreased Fe, C, and N content, while V element appears in the intermediate products.

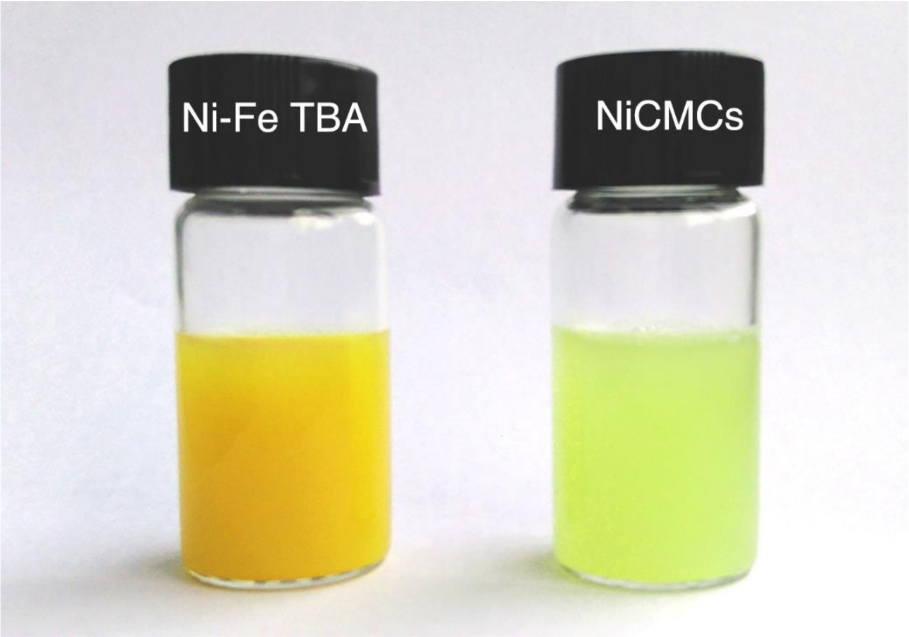

Fig. S7 Photographs of Ni-Fe TBA solid nanocube solution (left) and the NiCMCs solution (right)

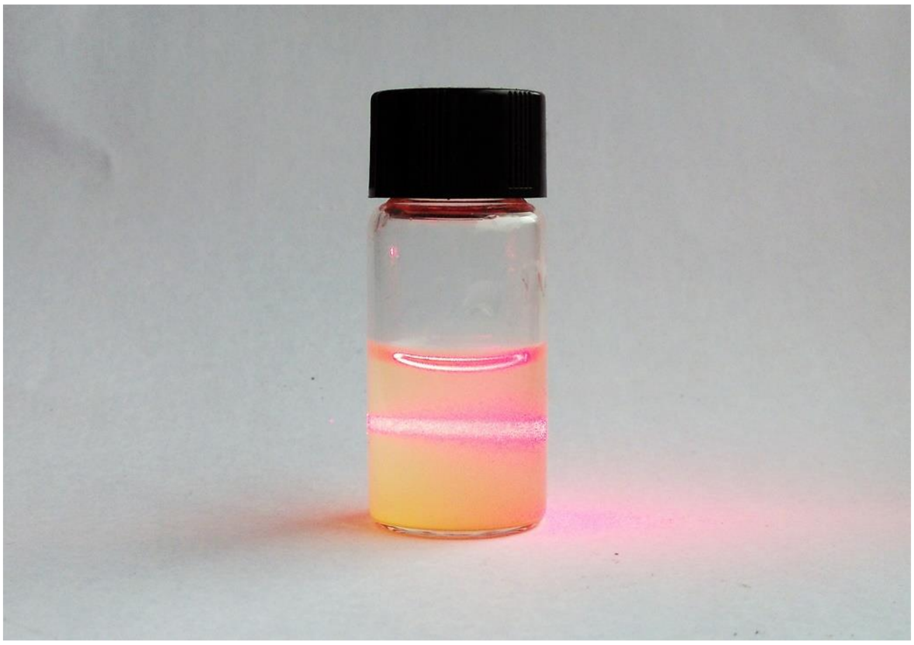

Fig. S8 Tyndall effect of NiCMCs solution. This indicates that NiCMCs can be uniformly dispersed in aqueous solution without aggregation or precipitation. It should be noted that in electrode materials, the crisscross assembly of ultrathin flakes makes the cubic cages stable, and the binder uniformly and stably fixes carbon black and cubic nanocages to the current collector surface, preventing them from easily dispersing in the electrolyte.

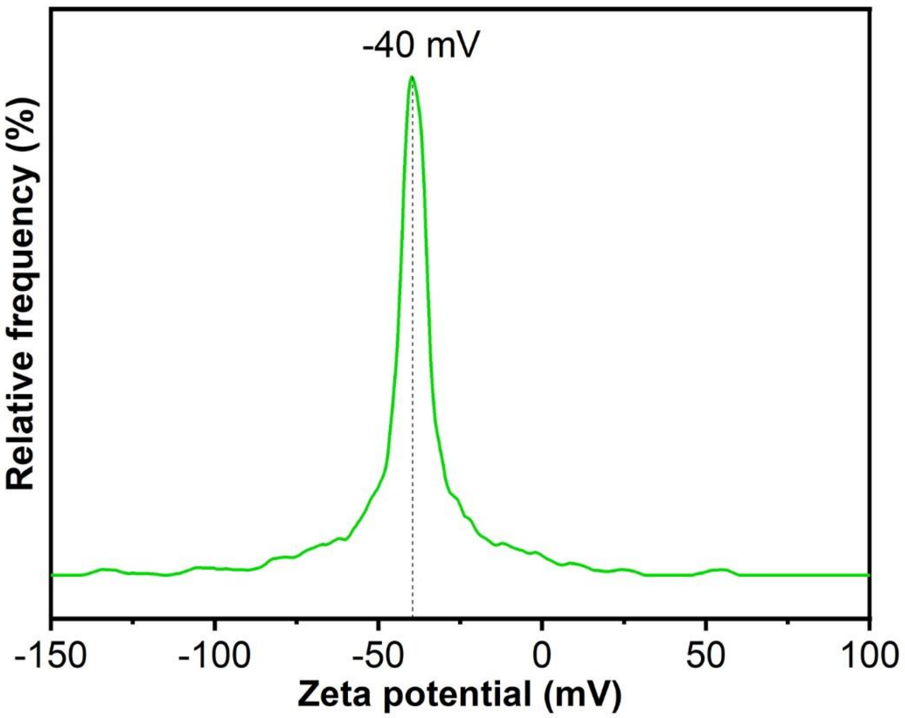

Fig. S9 Zeta potential of NiCMCs. The negative Zeta potential of approximately -40 mV confirms the negatively charged surface of NiCMCs, while the high potential value guarantees their stable existence in solution without aggregation.

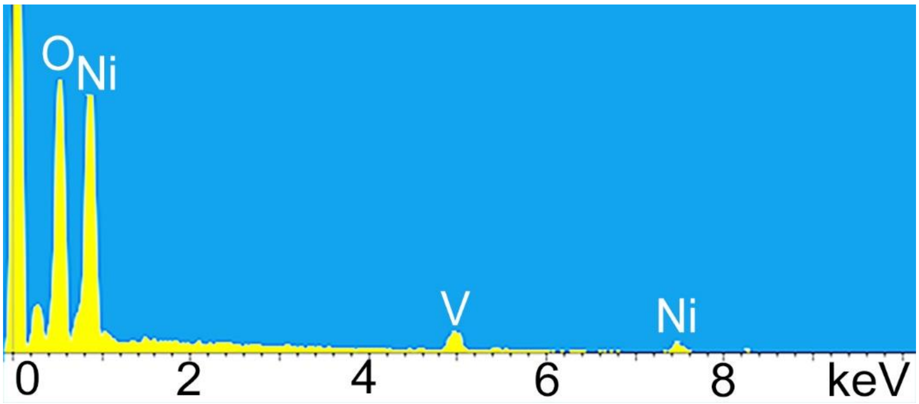

Fig. S10 EDS image of NiCMCs

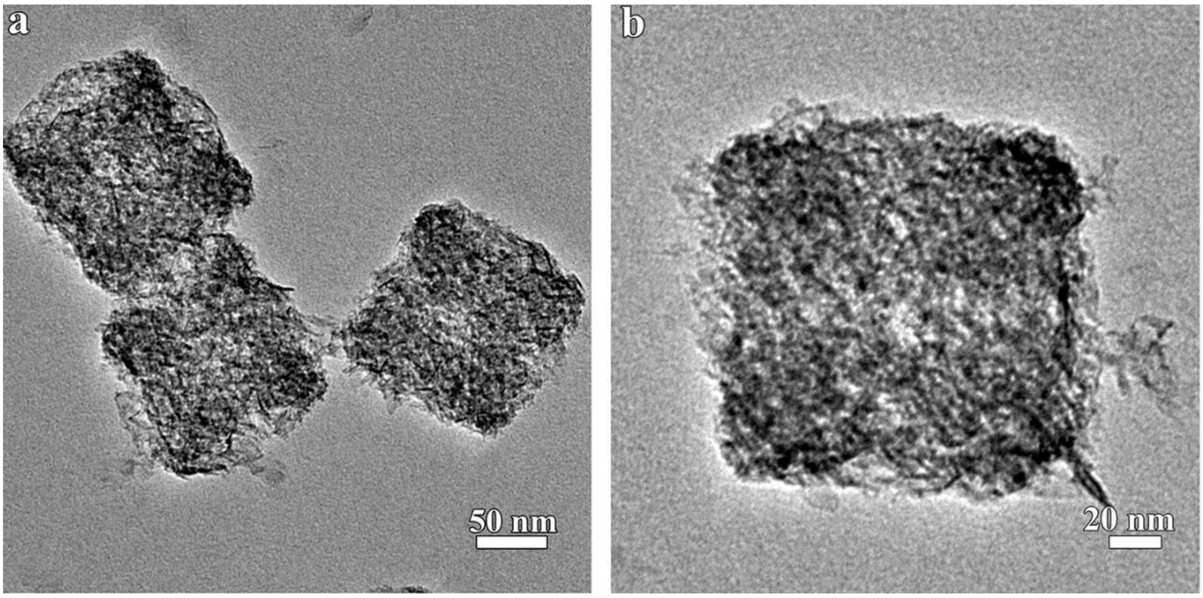

Fig. S11 TEM images of resulting $Ni(OH)_2$ using KOH as transformation reagent.

If Na_3VO_4 is not used in biomimetic transformation reactions, only particle-aggregated cubic structures can be obtained, which makes it difficult to achieve high atomic exposure and utilization ratio. This highlights the importance of biomimetic transformation synthesis to obtain NiCMCs with double-high properties.

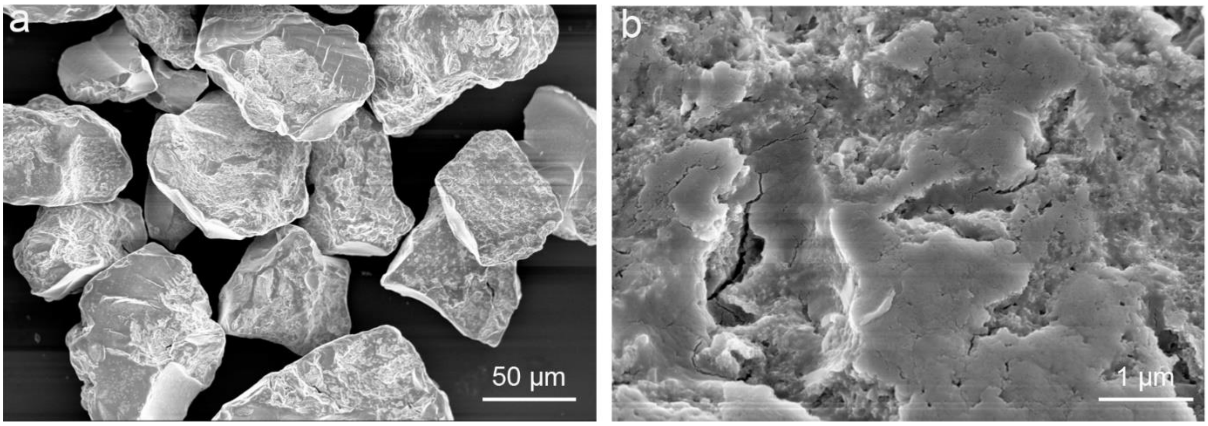

Fig. S12 SEM images of bulk $Ni(OH)_2$ at different magnifications.

By directly mixing a nickel acetate solution with a Na_3VO_4 solution, bulk $Ni(OH)_2$ was obtained. SEM results indicate that its morphology and size are completely different from our NiCMCs. The product is a large block-like structure with dimensions of approximately 50-100 microns, exhibiting a certain degree of porosity and roughness on its surface. This highlights the importance of biomimetic transformation synthesis in obtaining uniform high surface area materials.

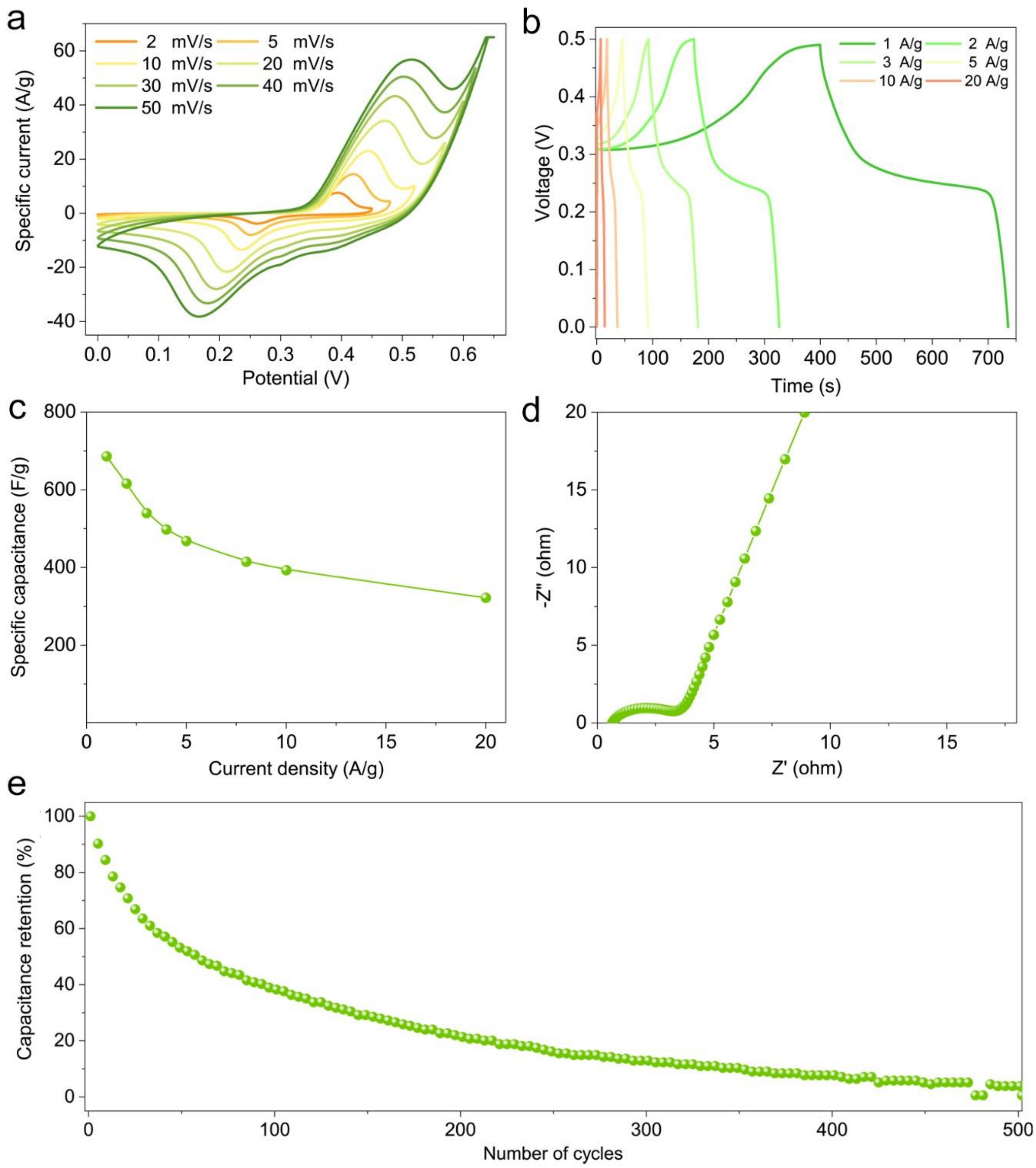

Fig. S13 Electrochemical properties of bulk $Ni(OH)_2$. (a) CV curves. (b) GCD curves. (c) Specific capacitances. (d) EIS curve. (e) Cycling property.

The bulk $Ni(OH)_2$ exhibits an electrochemical window similar to NiCMC, but its specific capacitance is only about 700 F/g. Moreover, its cyclic performance is poor; after 500 charge-discharge cycles, its specific capacitance is only about 5% of its initial value.

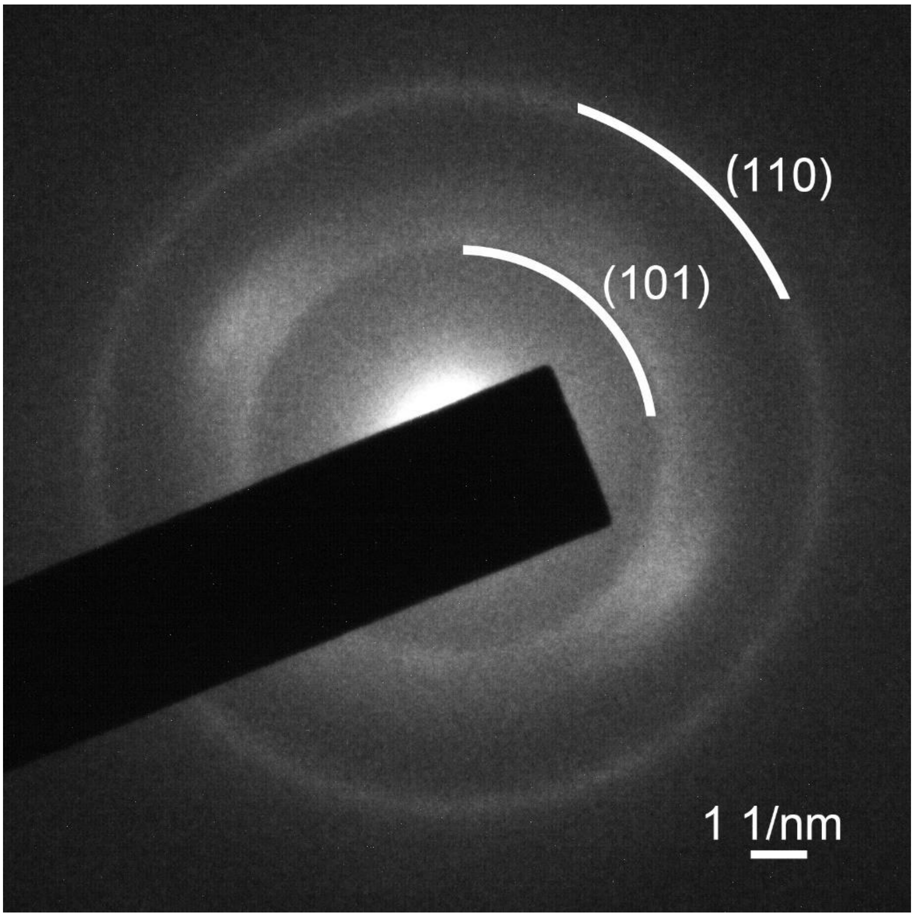

Fig. S14 Selected area electron diffraction (SAED) pattern of NiCMCs.

SAED pattern of NiCMCs exhibits two halos, indicating its polycrystalline structure and low degree of crystallinity.

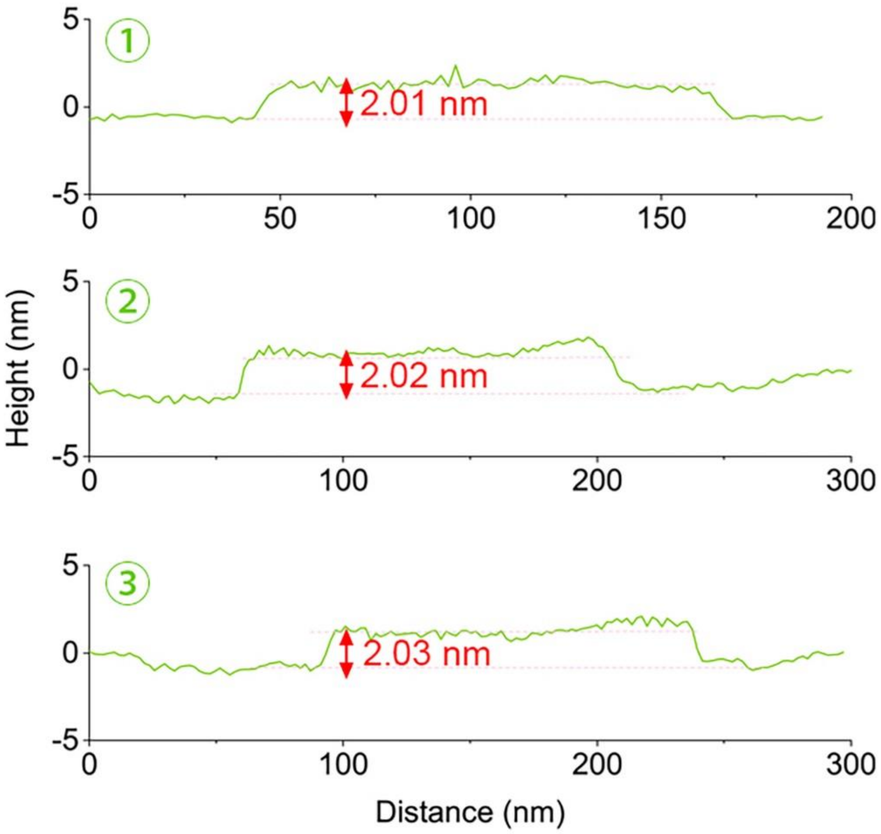

Fig. S15 Corresponding height profiles of NiCMCs in the AFM image.

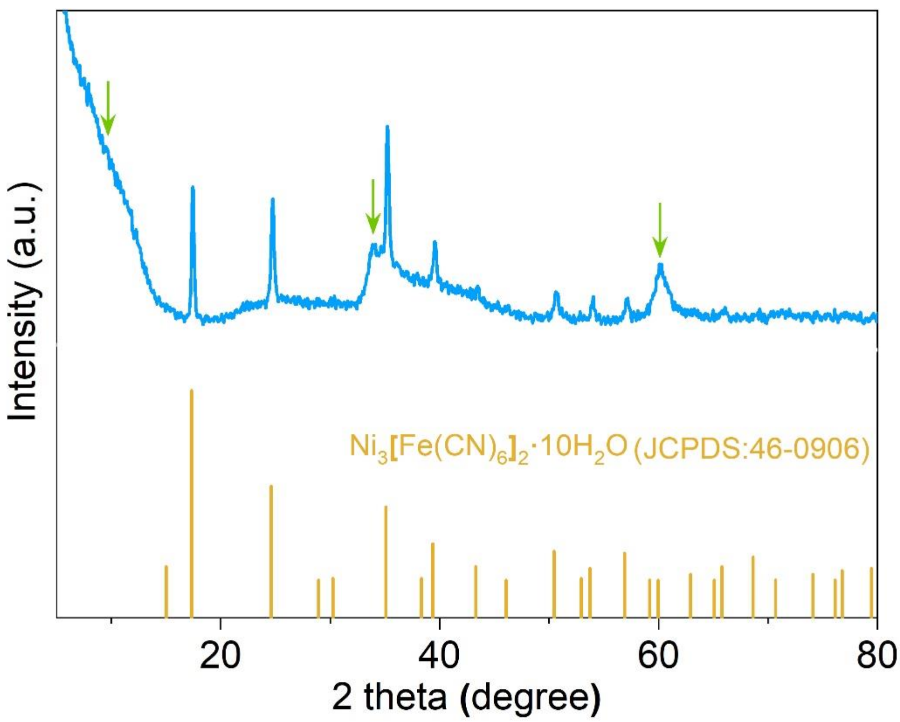

Fig. S16 XRD pattern of intermediate products during biomimetic transformation process.

The green arrows represent XRD diffraction peaks of α-$Ni(OH)_2$

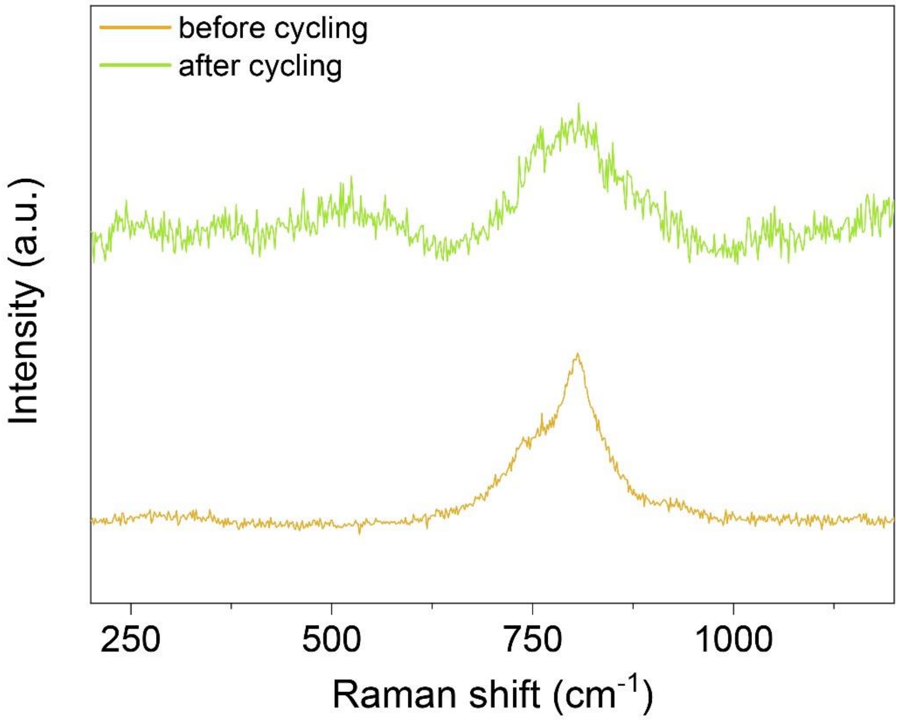

Fig. S17 Raman image of NiCMCs electrode before and after cycling.

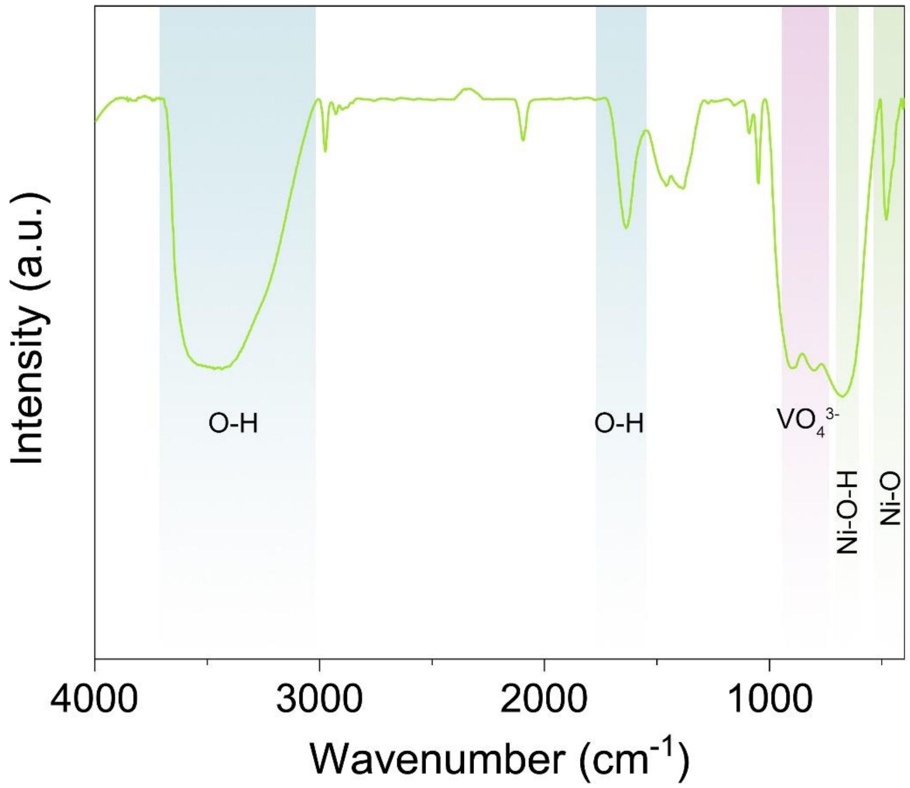

Fig. S18 FT-IR spectrum of NiCMCs.

The broad band observed at 3463 cm^{-1} corresponds to the O–H stretching mode, while the intense band at 1636 cm^{-1} represents the O–H bending mode of H_2O molecules in the interlayer. The band observed at 678 cm^{-1} is attributed to the Ni–O–H stretching mode, which is a characteristic mode of α-$Ni(OH)_2$. The stretching mode of Ni–O is confirmed by the band centered at 471 cm^{-1}. Additionally, the absorption bands at 798 cm^{-1} and 893 cm^{-1} are assigned to the asymmetric stretching and symmetric stretching of VO_4^{3-}.

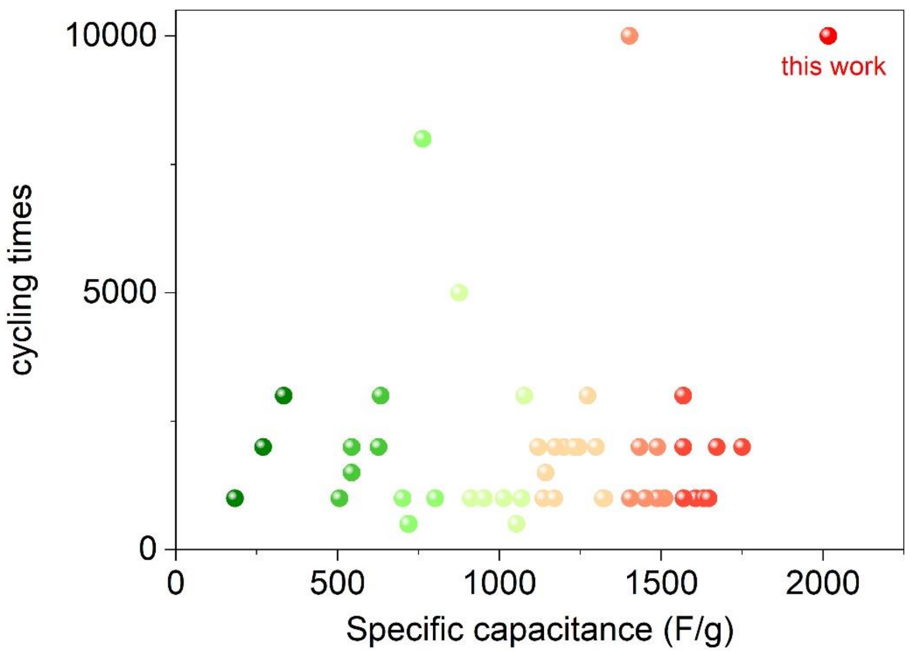

Fig. S19 The comparison of the reported $Ni(OH)_2$-based composites from the data of Table S3

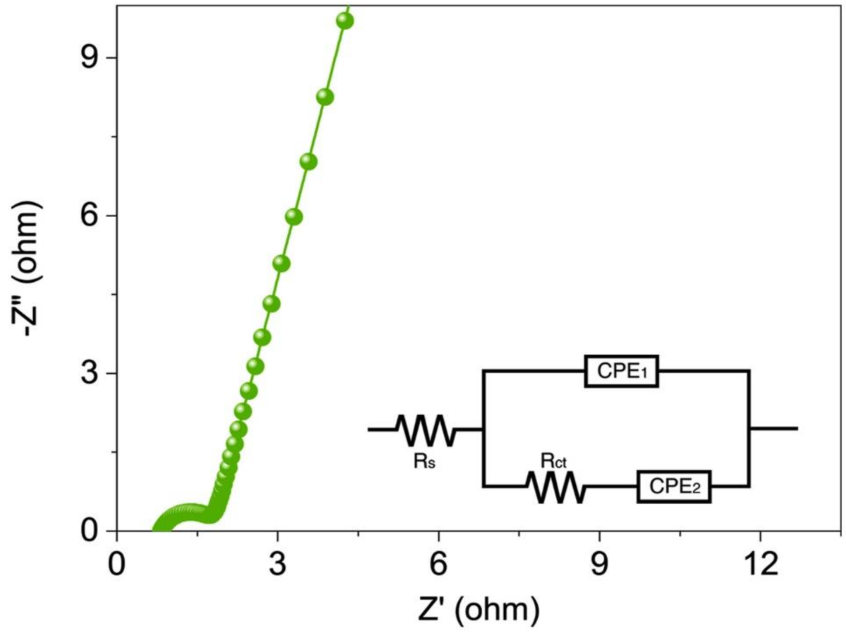

Fig. S20 EIS curve of NiCMCs in three-electrode system

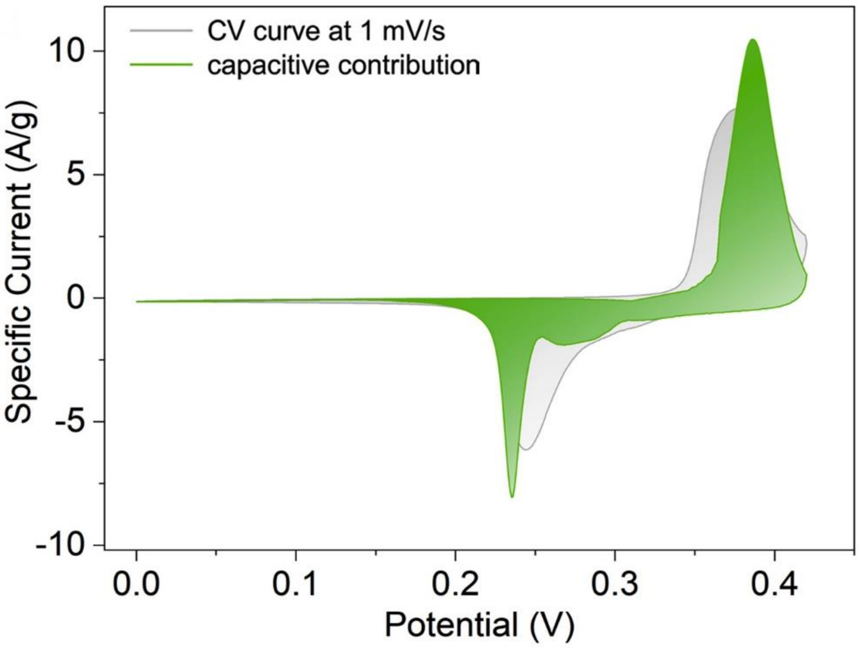

Fig. S21 Capacitive contribution of NiCMCs to charge storage at 1 mV/s

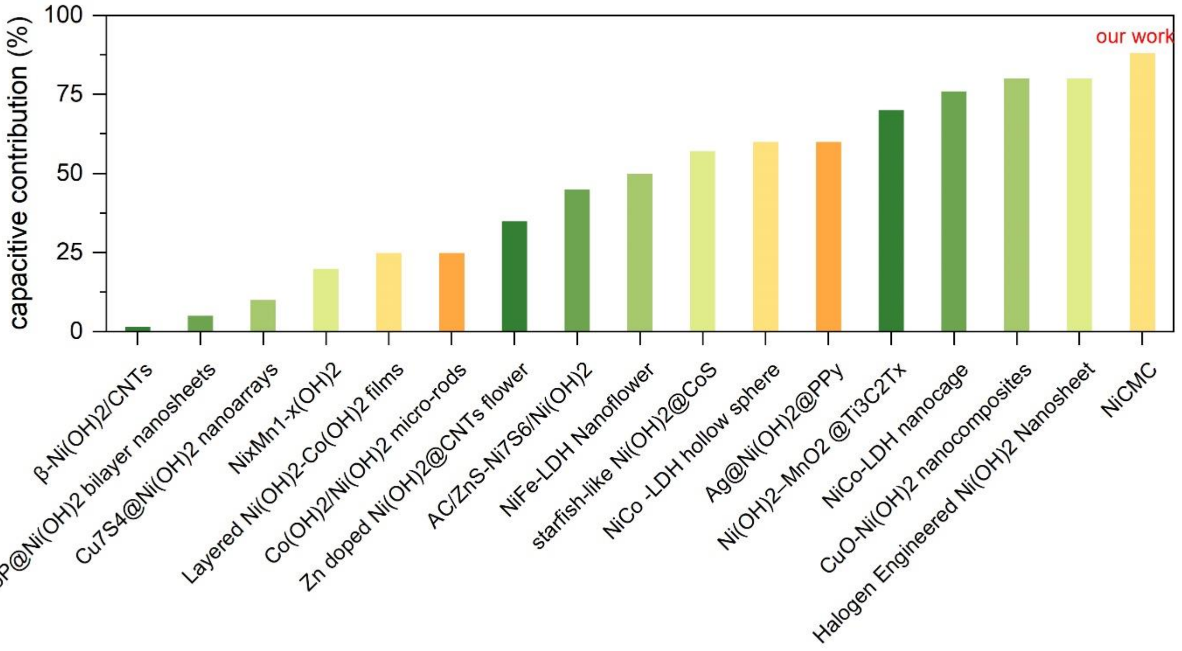

Fig. S22 Comparative capacitive contributions to charge storage of various reported $Ni(OH)_2$ based electrode materials[1-16]

References:

(1) Ren, X.; Gan, Z.; Sun, M.; Fang, Q.; Yan, Y.; Sun, Y.; Huang, J.; Cao, B.; Shen, W.; Li, Z.; et al. Colloidal synthesis of flower-like Zn doped $Ni(OH)_2$@CNTs at room-temperature for hybrid supercapacitor with high rate capability and energy density. *Electrochim. Acta* **2022**, *414*, 140208.

(2) Zhou, J.; Ji, W.; Xu, L.; Yang, Y.; Wang, W.; Ding, H.; Xu, X.; Wang, W.; Zhang, P.; Hua, Z.; et al. Controllable transformation of CoNi-MOF-74 on Ni foam into hierarchical-porous $Co(OH)_2/Ni(OH)_2$ micro-rods with ultra-high specific surface area for energy storage. *Chem. Eng. J.* **2022**, *428*, 132123.

(3) Chakrabarty, N.; Chakraborty, A. K. Controlling the electrochemical performance of β-$Ni(OH)_2$/carbon nanotube hybrid electrodes for supercapacitor applications by La doping: A systematic investigation. *Electrochim. Acta* **2019**, *297*, 173-187.

(4) Wang, J.; Yang, L.; Fu, Y.; Yin, P.; Guan, X.; Wang, G. Delicate control of crystallographic Cu2O derived Ni-Co amorphous double hydroxide nanocages for high-performance hybrid supercapacitors: an experimental and computational investigation. *Nanoscale* **2021**, *13*, 8562-8574.

(5) Wu, H.; Liu, M.; Liu, J.; Song, Y.; Sun, B.; Zhang, C.; Xu, Y.; Cao, Y.; Chen, C. Direct growth of AC/ZnS-$Ni_7S_6/Ni(OH)_2$ on nickel foam as a porous electrode material for high-performance supercapacitors. *Electrochim. Acta* **2023**, *441*, 141821.

(6) Mao, Y.; Xie, J.; Liu, H.; Hu, W. Hierarchical core-shell Ag@$Ni(OH)_2$@PPy nanowire electrode for ultrahigh energy density asymmetric supercapacitor. *Chem. Eng. J.* **2021**, *405*, 126984.

(7) Govindarajan, D.; Murugadoss, G.; Kirubaharan, K.; Manavalan, R. K.; Manibalan, G.; Shaikh, J.; Etesami, M.; Kheawhom, S. Improved electrochemical supercapacitive properties of CuO-$Ni(OH)_2$ nanocomposites by eco-friendly low-temperature synthesis. *J. Alloy. Compd*. **2023**, *942*, 169130.

(8) Tuyen, N.; Boudard, M.; Joao Carmezim, M.; Fatima Montemor, M. Layered $Ni(OH)_2$-$Co(OH)_2$ films prepared by electrodeposition as charge storage electrodes for hybrid supercapacitors. *Sci. Rep.* **2017**, *7*, 39980.

(9) Zhou, Y.; Zhao, S.; Yu, X.; Li, Y.; Chen, H.; Han, L. Metal-organic framework templated fabrication of Cu_7S_4@$Ni(OH)_2$ core-shell nanoarrays for high-performance supercapacitors. *Inorg. Chem. Front.* **2020**, *7*, 427-436.

(10) Hsu, S.; Chiang, H.; Huang, T.; Chao, S.; Wu, R. T.; Lu, C.; Huang, J.; Chang-Jian, C.; Weng, H. C.; Chen, H. Morphology evolution and electrochemical behavior of NixMn1-x$(OH)_2$ mixed hydroxides as high-performance electrode for supercapacitor. *Electrochim. Acta* **2022**, *403*, 139692.

(11) Li, Y.; He, G.; Huangfu, H.; Mi, Y.; Zhang, H.; Zheng, D.; Wu, M.; Yuan, H. Morphology evolution of NiFe layered double-hydroxide nanoflower clusters from nanosheets: controllable structure-performance relation for green energy storage. *Energy Technol.* **2024**, *12*, 2300749.

(12) Yang, Y.; Zhu, H.; Meng, H.; Ma, W.; Wang, C.; Ma, F.; Hu, Z. Nickel foam-supported starfish-like $Ni(OH)_2$@CoS nanostructure with obvious core-shell heterogeneous interfaces for hybrid supercapacitors application. *J. Mater. Sci.* **2021**, *56*, 3280-3295.

(13) Jiang, H.; Ke, Q.; Qiu, X.; Chen, J.; Chen, P.; Wang, S.; Luo, X.; Rao, B. NiCo layered double hydroxide nanocages for high-performance asymmetric supercapacitors. *Inorg. Chem. Front.* **2023**, *10*, 2154-2164.

(14) Liu, J.; Chen, Y.; Wang, Y.; Liu, L.; Chen, Q.; Shi, Q.; Huang, L.; Chen, X.; Xie, K. Rational design of CoP@$Ni(OH)_2$ bilayer nanosheets for high-performance supercapacitors. *New J. Chem.* **2023**, *47*, 7305-7311.

(15) Wu, W.; Liu, T.; Zhang, X.; Zhao, C.; Yi, D.; Fang, Y.; Diwu, J.; Wang, L.; Zhu, J. Rational design of hierarchical $Ni(OH)_2$-MnO_2 nanoflowers @$Ti_3C_2T_x$ nanosheets heterostructure as advanced symmetric supercapacitors. *J. Mater. Sci.-Mater. El.* **2023**, *34*, 855.

(16) Hao, J.; Yan, L.; Zou, X.; Bai, Y.; Han, Y.; Zhu, C.; Zhou, Y.; Xiang, B. Series of halogen engineered $Ni(OH)_2$ nanosheet for pseudocapacitive energy storage with high energy density. *Small* **2023**, *19*, 2300467.

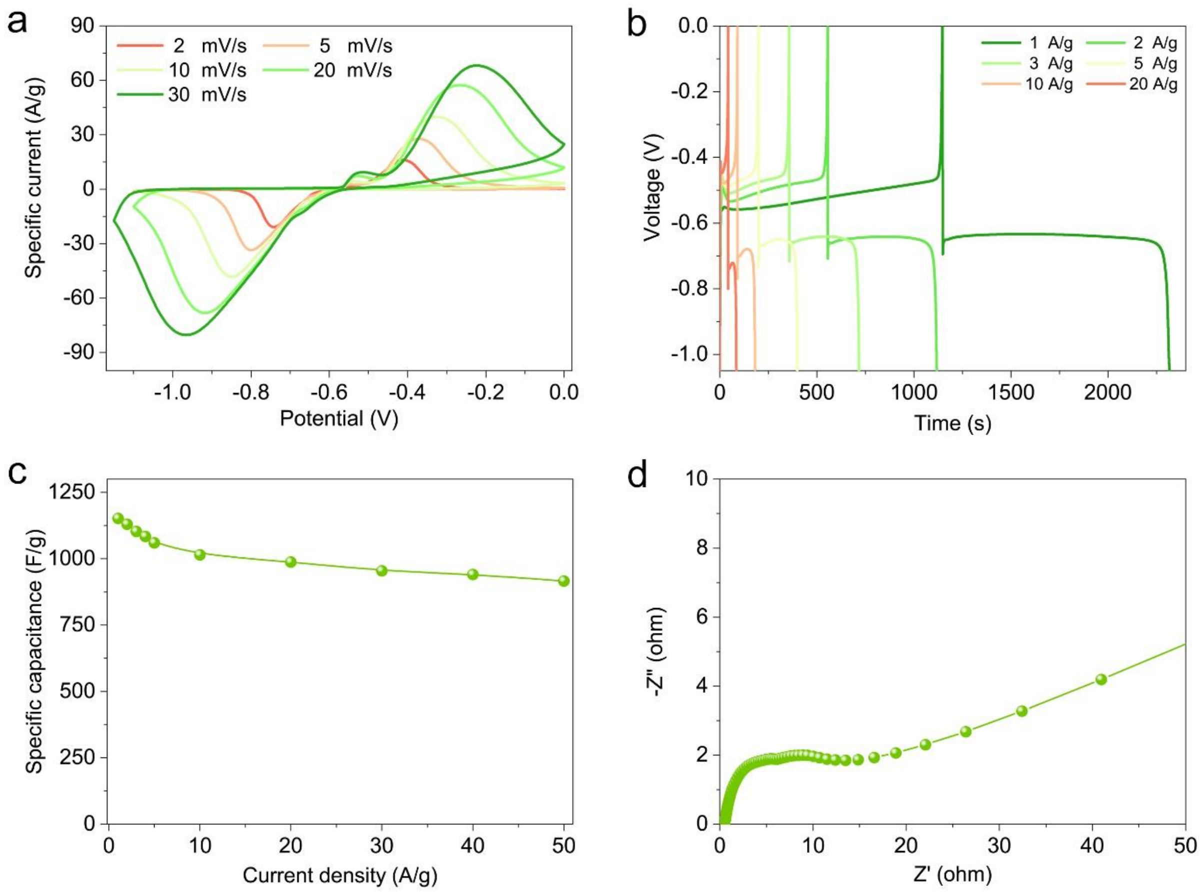

Fig. S23 Electrochemical properties of Bi_2O_3 in 6 M KOH using a three-electrode system. (a) CV curves. (b) GCD curves. (c) Specific capacitance values at different current densities. (d) EIS curve.

The electrochemical test results indicate that it has a potential window as wide as 1.2 V and a specific capacitance of up to 1150 F/g. Moreover, it also possesses excellent rate performance, as reflected in the GCD curves that display a charge/discharge current density of up to 50A/g. EIS tests show that its interfacial transfer resistance is small, providing support for its high capacity and superior rate capability. Thus, it can serve as an excellent anode electrode material to assemble hybrid energy storage devices with NiCMC.

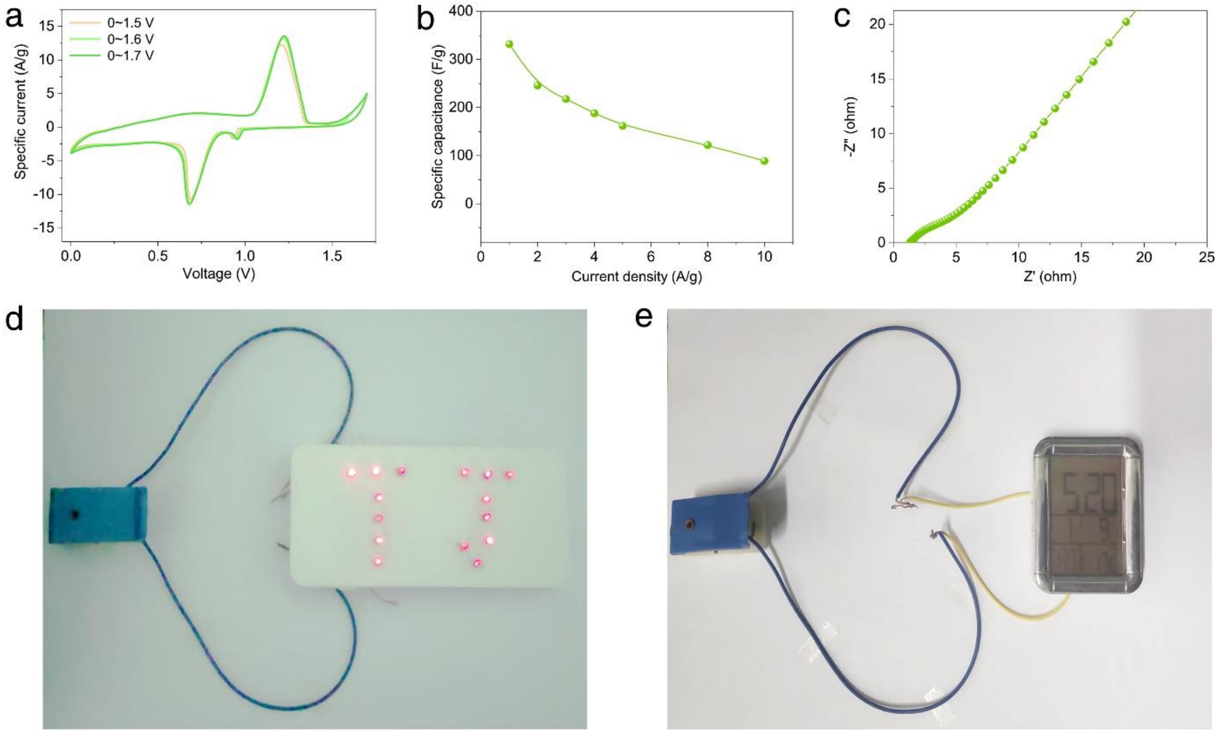

Fig. S24 Electrochemical properties of NiCMCs // Bi_2O_3 hybrid device. (a) CV curves at different potential windows. (b) Specific capacitance at different current densities. (c) EIS curve. (d, e) Photographs of the device lighting a LED array (d) and powering a digital electronic clock (e).

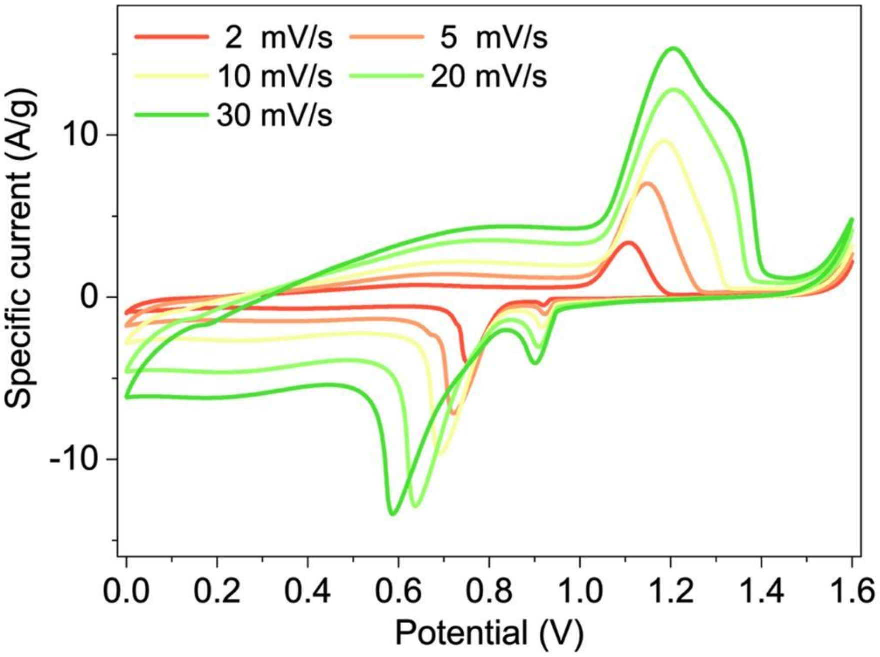

Fig. S25 CV curves of NiCMCs//Bi_2O_3 hybrid devices

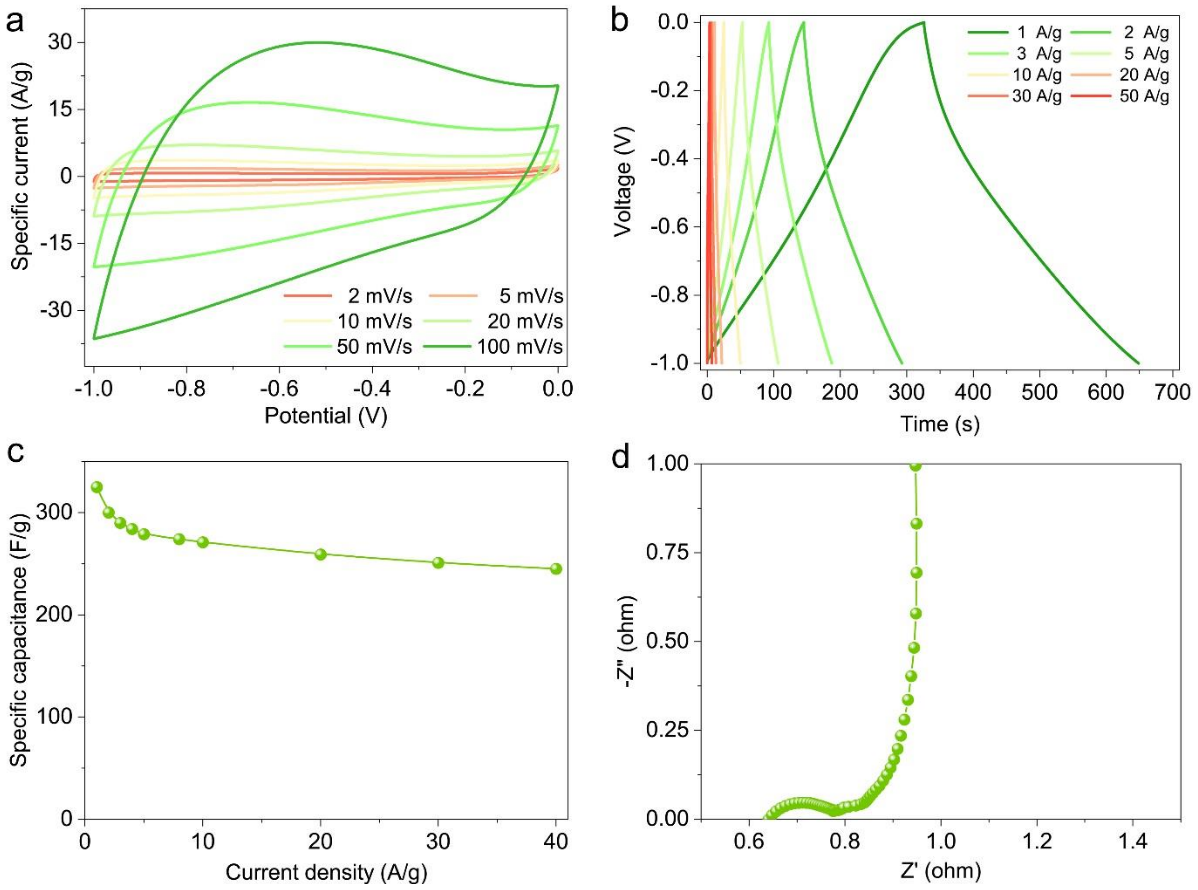

Fig. S26 Electrochemical properties of commercial porous carbon materials in 6 M KOH using a three-electrode system. (a) CV curves. (b) GCD curves. (c) Specific capacitance values at different current densities. (d) EIS curve.

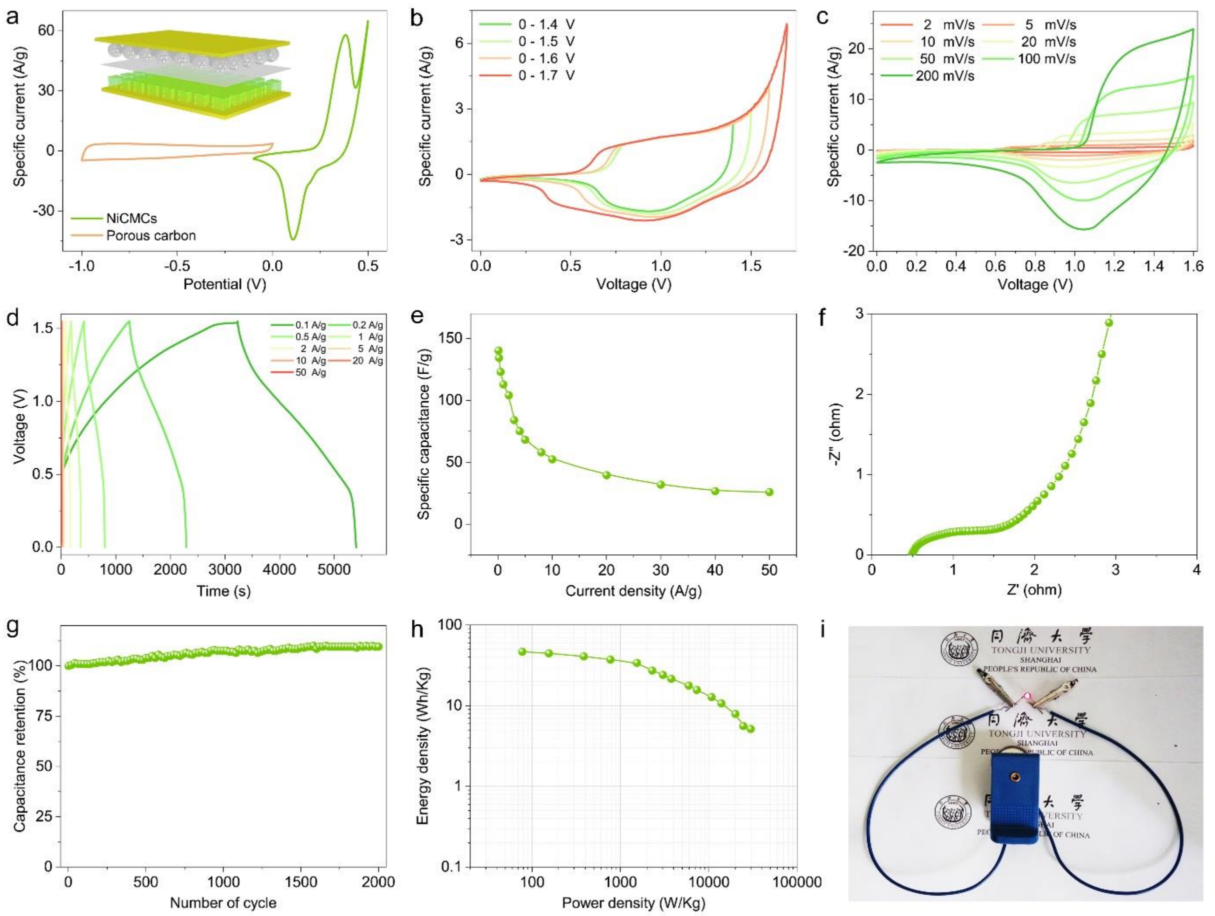

Fig. S27 Electrochemical properties of NiCMCs // porous carbon hybrid supercapacitor device. (a) CV curves of NiCMCs and porous carbon materials at the scan rate of 10 mV s^{-1}. (b) CV curves of device at different potential windows. (c) CV curves at different scan rates. (d) GCD curves at various current densities, (e) Specific capacitance of device at different current densities. (f) EIS curve. (g) Cycling property. (h) Ragone curve. (i) Photograph of the device lighting a LED.

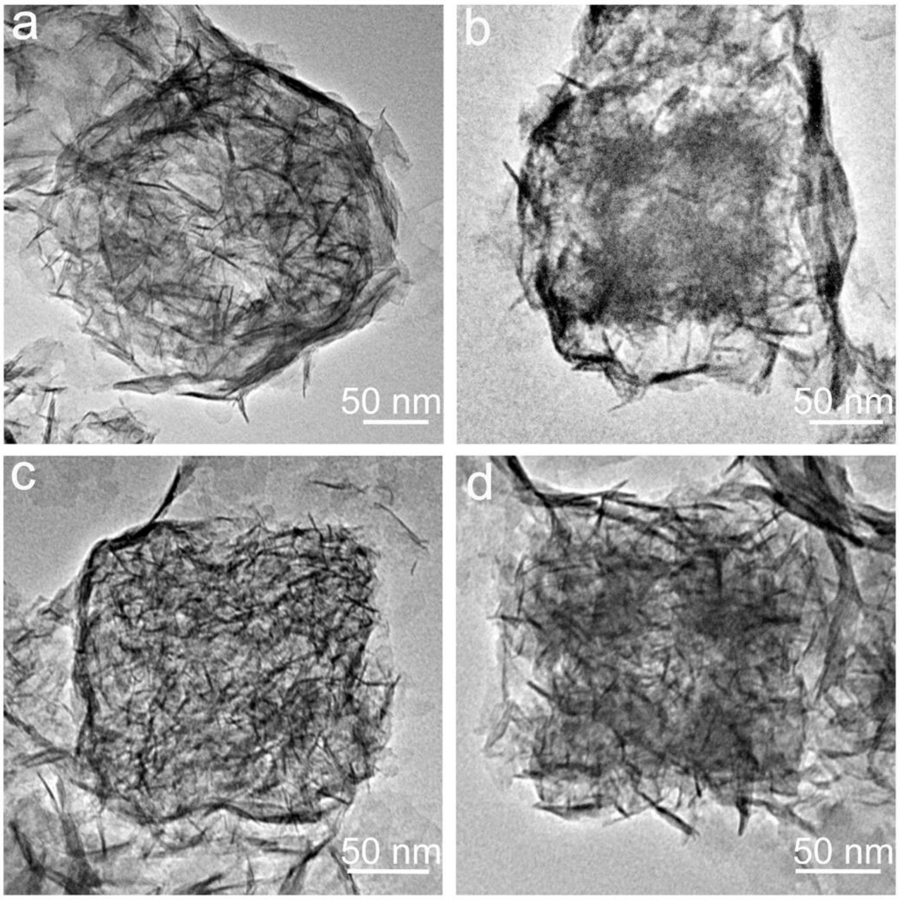

Fig. S28 ***ex-situ*** **TEM images of NiCMCs when charging to 0.45 V (a), charging to 0.5 V (b), discharging to 0.4 V (c), and discharging to 0 V (d).**

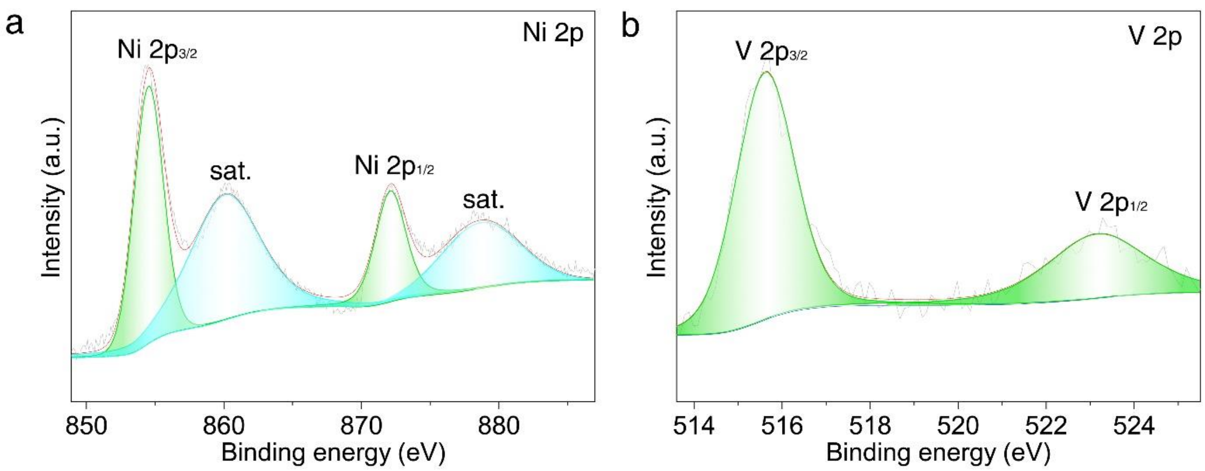

Fig. S29 XPS high-resolution spectra of NiCMCs after cycling.

Table S1 The specific surface area and porosity parameters of Ni-Fe TBA and NiCMCs

Samples	S_{BET} [m^2 g^{-1}]	$S_{langmuir}$ [m^2 g^{-1}]	V_{pore} [cm^3 g^{-1}]	V_{micro} [cm^3 g^{-1}]	D_{aver} [nm]
**Ni-Fe TBA nanocubes**	15	29	0.006	0.004	5.3
**NiCMCs**	505	735	1.1	0.29	6.6

S_{BET}: BET specific surface area

$S_{langmuir}$: Langmuir surface area

V_{pore}: Total pore volume

V_{micro}: Micropore volume

D_{aver}: Average pore size

Table S2 The comparison of the reported $Ni(OH)_2$ electrode materials

Materials	Specific surface area (m^2/g)	Specific capacitance (F/g)	Cycling (retention ratio)	Ref.
β-$Ni(OH)_2$ nanosphere	72.5	754.5 F/g (5 mV/s)	1000 (90.1%)	1
honey-comb like β- $Ni(OH)_2$ thin film	/	398 F/g (5 mV/s)	/	2
pompon-like β- $Ni(OH)_2$ hollow microspheres	39.2	1028.5 F/g (2.22 A/g)	1000	3
sphere-like $Ni(OH)_2$	131.92	982F/g (1A/g)	1000 (94%)	4
β- $Ni(OH)_2$ thin films	75	462 F/g (5 mV/s)	1000 (89%)	5
α- $Ni(OH)_2$ nanoparticles	/	534 F/g (2 mV/s)	/	6
β- $Ni(OH)_2$ particles	79	829 F/g (1 A/g)	1000 (60%)	7
amorphous $Ni(OH)_2$ nanoparticles	/	1460 F/g (2 mV/s)	1000 (70%)	8
Few-layered $Ni(OH)_2$ nanosheets	114	2064 F/g (2A/g)	/	9
$Ni(OH)_2$ nanoflake array	82.18	1747.5 F/g (1.02 mA/cm^2)	5000 (95.3%)	10
nanoporous $Ni(OH)_2$ thin-film	/	1519 F g (7 A/g)	10000 (90%)	11
sparse sheets	56.85	1200 F g (1 A/g)	/	12
Flower-like $Ni(OH)_2$	40.67	810.4 F g (1 A/g)	1000 (89%)	13
$Ni(OH)_2$ nanosheets on Ni foam		1540 F/g (1A/g)	500 (77%)	14
sphere-like $Ni(OH)_2$ nanofibers	286.4	1271 F/g (1A/g)	/	15
3D-connected α- $Ni(OH)_2$ sheets	126.9	549 C/g (2 A/g)	10000 (87.3%)	16
Flowerlike α- $Ni(OH)_2$	173	2030 F/g (1A/g)	1000 (83.4%)	17
mesoporous $Ni(OH)_2$ microsphere	101.3	1087 F/g (1A/g)	3000 (97.7%)	18
micro-belts like β- $Ni(OH)_2$ thin films	/	324 F/g (5 mV/s)	500 (78%)	19
nanosheets assembled prisms	274.29	830.6 C/g (0.5 A/g)	/	20
nanowires assembled α- $Ni(OH)_2$ microspheres	318	1610.8 F/g (0.5 A/g)	650	21
Porous $Ni(OH)_2$ Nanocube	194	1842 F/g (1A/g)	1000 (80.6%)	22
β- $Ni(OH)_2$ nanosphere	72.5	754.5 F/g (5 mV/s)	1000 (90.1%)	1
β- $Ni(OH)_2$ nanorod	91	1150 F/g (1 mv/s)	5000 (99%)	23
Cubic $Ni(OH)_2$ Nanocages	54.7	539.8 F/g (1 A/g)	2000 (96.9)	24
nanosheets assembled prisms	166.53	713.2 C/g (1 A/g)	1000 (~65%)	25
$Ni(OH)_2$ nanoplates	29.07	357 F/g (5 mV/s)	500 (71%)	26
α- $Ni(OH)_2$ nanospheres	119.4	1243 F/g (1A/g)	1500 (40%)	27
$Ni(OH)_2$ hollow spheres	199.2	700 F/g (1A/g)	2000 (50%)	28

Table S3 The comparison of the reported $Ni(OH)_2$-based composites

Materials	Specific capacitance (F/g)	Cycling (retention ratio)	Ref.
$Ni(OH)_2$/G/RGO	1510 F/g (2 A/g)	1000 (93.6%)	29
$Ni(OH)_2$-CNT composite films	544 F/g (10mV/s)	1500 (85%)	30
petal-like $Ni(OH)_2$/g-C_3N_4/RGO nanosheet	543.8 F/g (1 A/g)	2000 (72.7%)	31
CNT@$Ni(OH)_2$	1136 F/g (2 A/g)	1000 (92%)	32
$Ni(OH)_2$/Graphene	1735 F/g (1 mV/s)	/	33
$Ni(OH)_2$/AC/CNT composite	1038 F/g (1 A/g)	/	34
spherical $Ni(OH)_2$/CNTs	311 F/g	/	35
$Ni(OH)_2$ nanosheet arrays/biomiss-derived carbon microtubes	1568 F/g (1A/g)	3000 (84.3%)	36
flower-like Zn doped $Ni(OH)_2$@CNTs	750.5 C/g (0.5 A/g)	/	37
N-doped carbon spheres@$Ni(OH)_2$ nanocomposites	840 C/g (2 A/g)	2000 (75%)	38
Al-doped flower-like $Ni(OH)_2$	1750 F/g (1A/g)	2000 (72%)	39
$Ni(OH)_2$ Nanoparticles/MWCNTs	1487 F/g (5 Mv/s)	1000 (96%)	40
nano-petal $NI(OH)_2$/montmorillonite	1068 F/g (1A/g)	1000 (70.2%)	41
monolayer $Ni(OH)_2$ nanoflakes/3D graphene composites	1606 F/g (10A/g)	1000 (96%)	42
$Ni(OH)_2$/ MWCNTs	190 mAH/g (0.4A/g)	/	43
3D graphene/Ni $(OH)_2$	183.1 F/g	1000 (91.2%)	44
Ni-$Ni(OH)_2$/CNFs	763 F/g (1A/g)	8000 (94%)	45
expanded graphite/$Ni(OH)_2$	1569 F/g (1A/g)	1000 (73%)	46
$Ni(OH)_2$/graphene	1488 F/g (1 Mv/s)	2000 (90%)	47
carbon spheres@$Ni(OH)_2$	333.84 F/g (0.5 A/g)	3000 (75.34%)	48
$Ni(OH)_2$/CNTs nanoflake composites	720 F/g (1A/g)	500	49
carbon spheres@$Ni(OH)_2$	270.4 F/g (0.5A/g)	2000 (75.9%)	50
$Ni(OH)_2$/expanded graphite	1719.5 F/g (1A/g)	/	51
PANI-MWCNT-$Ni(OH)_2$	1013 F/g (1A/g)	1000 (75%)	52
$Ni(OH)_2$ nanoarray /RGO@carbon fabric	1325 F/g (1A/g)	1000 (90%)	53
$Ni(OH)_2$/graphene/bacterial cellulose paper	877.1 F/g	15000(93.6%)	54
vertically $Ni(OH)_2$ nanoplatelets on carbon nanofibers	701 F/g (5 mV/s)	1000 (83%)	55
Flower-like $Ni(OH)_2$ /g-C_3N_4	505.6 F/g (0.5 A/g)	1000 (75.1%)	56
$Ni(OH)_2$ hollow sphere@ Graphene	1368 F/g (1 A/g)	/	57
CNTs- $Ni(OH)_2$	912 F/g (10 mV/s)	1000 (84%)	58
rose-like $NI(OH)_2$/RGO	802 F/g (2A/g)	1000 (94.55%)	59
$Ni(OH)_2$ nanowall network@rGO	1402 F/g (5 mV/s)	10000 (73%)	60
$Ni(OH)_2$ nanoflakes/3D graphene foam	1450 F/g (5A/g)	1000(78%)	61
N-rGO/$Ni(OH)_2$	1077 (2A/g)	3000 (94%)	62
$Ni(OH)_2$/CNTs	876.5 F/g (0.5 A/g)	5000 (60%)	63

$Ni(OH)_2$/CNTs/$Ni(OH)_2$	1200 F/g (2A/g)	2000 (89%)	[64]
$Ni(OH)_2$/rGO	1671.67 F/g (1A/g)	2000 (81%)	[65]
$Ni(OH)_2$/sorghum stalk biomass carbon	889.2 F/g （2A/g）	30000 (95.9%)	[66]
La-decorated $Ni(OH)_2$ nanosheets	209.8 mAh/g (1 A/g)	/	[67]
$Ni(OH)_2$ nanocubes/GO	1299 F/g (0.5 A/g)	2000 (78%)	[68]
hexagonal beta-$Ni(OH)_2$/MWCNTs	1244 F/g (0.2 A/g)	2000 (66%)	[69]
$Ni(OH)_2$ Nanoflowers/Graphene Hydrogels	1632 F/g (1 A/g)	1000 (95.2%)	[70]
$Ni(OH)_2$/3D-rGO	1053.2 F/g (1A/g)	500 (92%)	[71]
$Ni(OH)_2$/RGO nanosheets	1143 F/g (1 A/g)	1500 (97%)	[72]
$Ni(OH)_2$ NPs/N-MWCNTs	350 F/g (0.5 A/g)	/	[73]
flower-like $Ni(OH)_2$/RGO nanosheets	618 C/g (3 A/g)	2000 (90%)	[74]
RGO/CNT/alpha-$Ni(OH)_2$	1320 F/g (6 A/g)	1000 (92.2%)	[75]
$Ni(OH)_2$ nanoflakes/CNT/rGO	633 F/g (1A/g)	3000 (80%)	[76]
$Ni(OH)_2$/rGO	1404 F/g (2A/g)	1000 (89.8%)	[77]
$Ni(OH)_2$@rGO NSs	1233 F/g (5 A/g)	2000	[78]
$Ni(OH)_2$ ultrathin sheets/rGO	580 C/g (1A/g)	5000(81%)	[79]
Amorphous $Ni(OH)_2$ nanobox/rGO	626.84 F/g (1A/g)	2000 (70%)	[80]
nanocrystalline $Ni(OH)_2$/MWCNTs	1466 F/g (5mV/S)	/	[81]
$Ni(OH)_2$-graphene sheet-carbon nanotube	1170.38 F/g (0.2A/g)	1000	[82]
rice-husk-derived porous carbon/$Ni(OH)_2$	952 F/g (1A/g)	1000 (81.3%)	[83]
Hexagonal $Ni(OH)_2$ nanoplates/graphene nanosheets	1172 F/g (5mV/s)	2000 (115%)	[84]
Standing $Ni(OH)_2$ Nanosheets/Multilayer Graphene	204.4 mAh/g (1A/g)	5000 (45.2%)	[85]
La-doped $Ni(OH)_2$	840 F/g (1A/g)	/	[86]
$Ni(OH)_2$ nanoplates/RGO nanofiber	1433 F/g (5mV/s)	2000 (90.5%)	[87]
3D marigold flower-like rGO/BN/$Ni(OH)_2$	349 F/g	/	[88]
$Ni(OH)_2$ nanosheets/ joint-welded carbon nanotube foam	1272 F/g (2A/g)	3000 (83.7%)	[89]
Ultrathin alpha-$Ni(OH)_2$ nanosheets	1568.3 F/g (1A/g)	2000	[90]
3D self-standing graphene/$Ni(OH)_2$	718.2 F/g (6.7 A/g)	500 (84.2%)	[91]
porous pompon-like Mg-incorporated alpha-$Ni(OH)_2$	1647 F/g	1000(82%)	[92]
3D $Ni(OH)_2$/rGO hydrogel	532 F/g	./	[93]
F-doped -$Ni(OH)_2$ mesoporous ultrathin nanosheets	1587.5 F/g	/	[94]
ultrathin $Ni(OH)_2$ nanosheets/rGO	1119.52 F/g	2000 (82.3%)	[95]

Table S4 Element contents determined by ICP

**Elements**	**wt.%**
Ni	61.4895%
V	0.7205%

References:

(1) Tizfahm, J.; Safibonab, B.; Aghazadeh, M.; Majdabadi, A.; Sabour, B.; Dalvand, S. Supercapacitive behavior of beta-$Ni(OH)_2$ nanospheres prepared by a facile electrochemical method. *Colloid. Surface. A.* **2014**, *443*, 544-551.

(2) Patil, U. M.; Gurav, K. V.; Fulari, V. J.; Lokhande, C. D.; Joo, O. S. Characterization of honeycomb-like beta-$Ni(OH)_2$ thin films synthesized by chemical bath deposition method and their supercapacitor application. *J. Power Sources* **2009**, *188*, 338-342.

(3) Wang, Y.; Gai, S.; Li, C.; He, F.; Zhang, M.; Yan, Y.; Yang, P. Controlled synthesis and enhanced supercapacitor performance of uniform pompon-like beta-$Ni(OH)_2$ hollow microspheres. *Electrochim. Acta* **2013**, *90*, 673-681.

(4) Tang, Q.; Ma, L.; Cao, F.; Gan, M.; Yan, F. Different morphologies of $Ni(OH)_2$ derived from a mof template for high performance supercapacitors. *J. Mater. Sci.-Mater. El.* **2019**, *30*, 9114-9122.

(5) Dubal, D. P.; Fulari, V. J.; Lokhande, C. D. Effect of morphology on supercapacitive properties of chemically grown beta- $Ni(OH)_2$ thin films. *Micropor. Mesopor. Mat.* **2012**, *151*, 511-516.

(6) Vijayakumar, S.; Muralidharan, G. Electrochemical supercapacitor behaviour of alpha-$Ni(OH)_2$ nanoparticles synthesized via green chemistry route. *J. Electroanal. Chem.* **2014**, *727*, 53-58.

(7) Chen, L.; Yang, X.; Tian, Y.; Wang, Y.; Zhao, X.; Lei, X.; Zhang, F. Fabrication of beta- $Ni(OH)_2$ particles by alkaline etching layered double hydroxides precursor for supercapacitor. *Front. Energy Res.* **2022**, *9*, 810568.

(8) Hu, L.; Yu, Z.; Hu, Z.; Song, Y.; Zhang, F.; Zhu, H.; Jiao, S. Facile synthesis of amorphous $Ni(OH)_2$ for high-performance supercapacitors via electrochemical assembly in a reverse micelle. *Electrochim. Acta* **2015**, *174*, 273-281.

(9) Sun, W.; Rui, X.; Ulaganathan, M.; Madhavi, S.; Yan, Q. Few-layered $Ni(OH)_2$ nanosheets for high-performance supercapacitors. *J. Power Sources* **2015**, *295*, 323-328.

(10) Song, D.; Zhu, J.; Xuan, L.; Zhao, C.; Xie, L.; Chen, L. Freestanding two-dimensional $Ni(OH)_2$ thin sheets assembled by 3D nanoflake array as basic building units for supercapacitor electrode materials. *J. Colloid Interf. Sci.* **2018**, *509*, 163-170.

(11) Yang, Y.; Li, L.; Ruan, G.; Fei, H.; Xiang, C.; Fan, X.; Tour, J. M. Hydrothermally formed three-dimensional nanoporous $Ni(OH)_2$ thin-film supercapacitors. *ACS Nano* **2014**, *8*, 9622-9628.

(12) Liang, R.; Wang, G.; Huang, X.; Zhu, L.; Li, S.; Yan, Y.; Zhong, B. Improving the specific capacitance of $Ni(OH)_2$-based supercapacitors by tailoring its porous structures and particle size. *Mater. Lett.* **2015**, *158*, 128-131.

(13) Zhang, Y.; Liu, Y.; Guo, Y.; Yeow, Y. X.; Duan, H.; Li, H.; Liu, H. In situ preparation of flower-like alpha-$Ni(OH)_2$ and NiO from nickel formate with excellent capacitive properties as electrode materials for supercapacitors. *Mater. Chem. Phys.* **2015**, *151*, 160-166.

(14) Cheng, X.; Zhang, D.; Liu, X.; Cao, D.; Wang, G. Influence of CTAB on morphology, structure, and supercapacitance of beta-$Ni(OH)_2$. *Ionics* **2015**, *21*, 533-540.

(15) Li, W.; Yang, L.; Lin, B.; Isimjan, T. T.; Yang, D.; Hu, Y.; Hu, Z.; Sacher, E. Large-scale synthesis of 3D sphere-like hierarchical $Ni(OH)_2$ nanofibers for high-performance electrochemical supercapacitors. *Mater. Res. Express* **2015**, *2*, 095008.

(16) William, J. J.; Babu, I. M.; Muralidharan, G. Microwave assisted fabrication of L-arginine capped alpha-$Ni(OH)_2$ microstructures as an electrode material for high performance hybrid supercapacitors. *Mater. Chem. Phys.* **2019**, *224*, 357-368.

(17) Zhang, X.; Li, C.; Miao, W.; Sun, X.; Wang, K.; Ma, Y. Microwave-assisted synthesis of 3D flowerlike alpha-$Ni(OH)_2$ nanostructures for supercapacitor application. *Sci. China Technol. Sc.* **2015**, *58*, 1871-1876.

(18) Wang, D.; Guan, B.; Li, Y.; Li, D.; Xu, Z.; Hu, Y.; Wang, Y.; Zhang, H. Morphology-controlled synthesis of hierarchical mesoporous alpha-$Ni(OH)_2$ microspheres for high-performance asymmetric supercapacitors. *J. Alloy. Compd* **2018**, *737*, 238-247.

(19) Gund, G. S.; Dubal, D. P.; Shinde, S. S.; Lokhande, C. D. One step hydrothermal synthesis of micro-belts like beta-$Ni(OH)_2$ thin films for supercapacitors. *Ceram. Int.* **2013**, *39*, 7255-7261.

(20) Zhang, H.; Xu, B.; Xiao, Z.; Mei, H.; Zhang, L.; Han, Y.; Sun, D. Optimizing crystallinity and porosity of hierarchical $Ni(OH)_2$ through conformal transformation of metal-organic framework template for supercapacitor applications. *Crystengcomm* **2018**, *20*, 4313-4320.

(21) Du, H.; Jiao, L.; Cao, K.; Wang, Y.; Yuan, H. Polyol-mediated synthesis of mesoporous alpha-$Ni(OH)_2$ with enhanced supercapacitance. *ACS Appl. Mater. Inter.* **2013**, *5*, 6643-6648.

(22) Li, L.; Tan, L.; Li, G.; Zhang, Y.; Liu, L. Self-templated synthesis of porous $Ni(OH)_2$ nanocube and its high electrochemical performance for supercapacitor. *Langmuir* **2017**, *33*, 12087-12094.

(23) Lakshmi, V.; Ranjusha, R.; Vineeth, S.; Nair, S. V.; Balakrishnan, A. Supercapacitors based on microporous beta-$Ni(OH)_2$ nanorods. *Colloid. Surface. A.* **2014**, *457*, 462-468.

(24) Tian, L.; Yang, T.; Pu, W.; Zhang, J. Synthesis of cubic $Ni(OH)_2$ nanocages through coordinating etching and precipitating route for high-performance supercapacitors. *Nanoscale Res. Lett.* **2019**, *14*, 1-9.

(25) Zhang, S.; Yang, Z.; Gong, K.; Xu, B.; Mei, H.; Zhang, H.; Zhang, J.; Kang, Z.; Yan, Y.; Sun, D. Temperature controlled diffusion of hydroxide ions in 1d channels of Ni-MOF-74 for its complete conformal hydrolysis to hierarchical $Ni(OH)_2$ supercapacitor electrodes. *Nanoscale* **2019**, *11*, 9598-9607.

(26) Gund, G. S.; Dubal, D. P.; Jambure, S. B.; Shinde, S. S.; Lokhande, C. D. Temperature influence on morphological progress of $Ni(OH)_2$ thin films and its subsequent effect on electrochemical supercapacitive properties. *J. Mater Chem A* **2013**, *1*, 4793-4803.

(27) Zhang, R.; Tu, Q.; Li, X.; Sun, X.; Liu, X.; Chen, L. Template-free preparation of alpha-$Ni(OH)_2$ nanosphere as high-performance electrode material for advanced supercapacitor. *Nanomaterials* **2022**, *12*.

(28) Sun, H.; Liu, S.; Lu, Q.; Zhong, H. Template-synthesis of hierarchical $Ni(OH)_2$ hollow spheres with excellent performance as supercapacitor. *Mater. Lett.* **2014**, *128*, 136-139.

(29) Tian, J.; Shan, Q.; Yin, X.; Wu, W. A facile preparation of graphene/reduced graphene oxide/$Ni(OH)_2$ two dimension nanocomposites for high performance supercapacitors. *Adv. Powder Technol.* **2019**, *30*, 3118-3126.

(30) Abitkar, S. B.; Jadhav, P. R.; Tarwal, N. L.; Moholkar, A. V.; Patil, C. E. A facile synthesis of alpha-$Ni(OH)_2$-CNT composite films for supercapacitor application. *Adv. Powder Technol.* **2019**, *30*, 2285-2292.

(31) Liu, H.; Liu, B.; Sun, X.; Han, X.; Cui, J.; Zhang, Y.; He, W. A simple hydrothermal method for the preparation of 3D petal-like $Ni(OH)_2$/g-c3n4/rGO composite with good supercapacitor performance. *Inorg. Chem. Commun.* **2020**, *122*, 108263.

(32) Yi, H.; Wang, H.; Jing, Y.; Peng, T.; Wang, Y.; Guo, J.; He, Q.; Guo, Z.; Wang, X. Advanced asymmetric supercapacitors based on CNT@$Ni(OH)_2$ core-shell composites and 3D graphene networks. *J. Mater. Chem. A* **2015**, *3*, 19545-19555.

(33) Shahid, M.; Liu, J.; Shakir, I.; Warsi, M. F.; Nadeem, M.; Kwon, Y. Facile approach to synthesize $Ni(OH)_2$ nanoflakes on MWCNTs for high performance electrochemical supercapacitors. *Electrochim. Acta* **2012**, *85*, 243-247.

(34) Sui, L.; Tang, S.; Chen, Y.; Dai, Z.; Huangfu, H.; Zhu, Z.; Qin, X.; Deng, Y.; Haarberg, G. M. An asymmetric supercapacitor with good electrochemical performances based on $Ni(OH)_2$/AC/CNT and AC. *Electrochim. Acta* **2015**, *182*, 1159-1165.

(35) Wang, X.; Ruan, D.; You, Z. Application of spherical $Ni(OH)_2$/CNTs composite electrode in FP asymmetric supercapacitor. *T. Nonferr. Metal. Soc.* **2006**, *16*, 1129-1134.

(36) Li, Q.; Lu, C.; Xiao, D.; Zhang, H.; Chen, C.; Xie, L.; Liu, Y.; Yuan, S.; Kong, Q.; Zheng, K.; et al. Beta-$Ni(OH)_2$ nanosheet arrays grown on biomass-derived hollow carbon microtubes for high-performance asymmetric supercapacitors. *Chemelectrochem* **2018**, *5*, 1279-1287.

(37) Ren, X.; Gan, Z.; Sun, M.; Fang, Q.; Yan, Y.; Sun, Y.; Huang, J.; Cao, B.; Shen, W.; Li, Z.; et al. Colloidal synthesis of flower-like Zn doped $Ni(OH)_2$@CNTs at room-temperature for hybrid supercapacitor with high rate capability and energy density. *Electrochim. Acta* **2022**, *414*, 140208.

(38) Zhang, L.; Li, G.; Jing, L.; Li, Z.; Li, Z.; Yao, H.; Wang, J.; Liu, Q.; Han, Y. Controllable and fast growth of ultrathin alpha-$Ni(OH)_2$ nanosheets on polydopamine based N-doped carbon spheres for supercapacitors application. *Synthetic Met.* **2020**, *270*, 116580.

(39) Wei, J.; Qiu, D.; Li, M.; Xie, Z.; Gao, A.; Liu, H.; Yin, S.; Yang, D.; Yang, R. Controllable synthesis of aluminum doped peony-like -$Ni(OH)_2$ with ultrahigh rate capability for asymmetric supercapacitors. *RSC Adv* **2019**, *9*, 10237-10244.

(40) Dubal, D. P.; Gund, G. S.; Lokhande, C. D.; Holze, R. Decoration of spongelike Ni(OH)2 nanoparticles onto MWCNTs using an easily manipulated chemical protocol for supercapacitors. *ACS Appl Mater Inter* **2013**, *5*, 2446-2454.

(41) Xu, G. M.; Wang, M.; Bao, H. L.; Fang, P. F.; Zeng, Y. H.; Du, L.; Wang, X. L. Design of $Ni(OH)_2$/M-MMT nanocomposite with higher charge transport as a high capacity supercapacitor. *Front. Chem.* **2022**, *10*, 916860.

(42) Lan, W.; Tang, G.; Sun, Y.; Wei, Y.; La, P.; Su, Q.; Xie, E. Different-layered $Ni(OH)_2$ nanoflakes/3D graphene composites for flexible supercapacitor electrodes. *J. Mater. Sci.-Mater. El.* **2016**, *27*, 2741-2747.

(43) Wang, Y. G.; Yu, L.; Xia, Y. Y. Electrochemical capacitance performance of hybrid supercapacitors based on $Ni(OH)_2$/carbon nanotube composites and activated carbon. *J. Electrochem. Soc.* **2006**, *153*, A743-A748.

(44) Yan, H.; Bai, J.; Wang, B.; Yu, L.; Zhao, L.; Wang, J.; Liu, Q.; Liu, J.; Li, Z. Electrochemical reduction approach-based 3D graphene/$Ni(OH)_2$ electrode for high-performance supercapacitors. *Electrochim. Acta* **2015**, *154*, 9-16.

(45) Erdemutu, E.; Bai, C.; Ding, L. Electrospun Ni-$Ni(OH)_2$/carbon nanofibers as flexible binder-free supercapacitor electrode with enhanced specific capacitance. *J. Electron. Mater.* **2020**, *49*, 7211-7218.

(46) Qu, R.; Tang, S.; Qin, X.; Yuan, J.; Deng, Y.; Wu, L.; Li, J.; Wei, Z. Expanded graphite supported $Ni(OH)_2$ composites for high performance supercapacitors. *J. Alloy. Compd* **2017**, *728*, 222-230.

(47) Shen, P.; Zhang, H.; Zhang, S.; Fei, L. Fabrication of completely interface-engineered $Ni(OH)_2$/rGO nanoarchitectures for high-performance asymmetric supercapacitors. *Appl. Surf. Sci.* **2018**, *460*, 65-73.

(48) Mao, Z.; Zhou, Y.; Wang, Z.; Yang, Z.; Liu, X. Fabrication of sponge-like alpha-$Ni(OH)_2$ on styrene-acrylonitrile copolymer (san)-derived carbon spheres as electrode materials for supercapacitor application. *RSC Adv* **2016**, *6*, 100623-100631.

(49) Cheng, H.; Su, A. D.; Li, S.; Nguyen, S. T.; Lu, L.; Lim, C. Y. H.; Duong, H. M. Facile synthesis and advanced performance of $Ni(OH)_2$/CNTs nanoflake composites on supercapacitor applications. *Chem. Phys. Lett.* **2014**, *601*, 168-173.

(50) Le, Q.; Wang, T.; Zhu, S.; Zhang, J.; Zhang, Y. Facile synthesis of carbon sphere@$Ni(OH)_2$ and derivatives for high-performance supercapacitors. *Funct. Mater. Lett.* **2016**, *9*, 1642002.

(51) Yuan, J.; Tang, S.; Zhu, Z.; Qin, X.; Qu, R.; Deng, Y.; Wu, L.; Li, J.; Haarberg, G. M. Facile synthesis of high-performance $Ni(OH)_2$/expanded graphite electrodes for asymmetric supercapacitors. *J. Mater. Sci.-Mater. El.* **2017**, *28*, 18022-18030.

(52) Thomas, L.; Pete, S.; Chaitra, K.; Venkatesh, K.; Gopalkrishna, B.; Nagaraju, K. Facile synthesis of PANI-MWCNT-$Ni(OH)_2$ ternary composites and study of their performance as electrode material for supercapacitors. *Diam. Relat. Mater.* **2020**, *106*, 107853.

(53) Liu, X.; Du, S.; Zuo, X.; Zhang, X.; Jiang, Y. Facile synthesis of $Ni(OH)_2$ nanoarrays on graphene@carbon fabric as dual-functional electrochemical materials for supercapacitors and capacitive desalination. *RSC Adv* **2021**, *12*, 1177-1183.

(54) Ma, L.; Liu, R.; Liu, L.; Wang, F.; Niu, H.; Huang, Y. Facile synthesis of $Ni(OH)_2$/graphene/bacterial cellulose paper for large areal mass, mechanically tough and flexible supercapacitor electrodes. *J. Power Sources* **2016**, *335*, 76-83.

(55) Zhang, L.; Ding, Q.; Huang, Y.; Gu, H.; Miao, Y.; Liu, T. Flexible hybrid membranes with $Ni(OH)_2$ nanoplatelets vertically grown on electrospun carbon nanofibers for high-performance supercapacitors. *ACS Appl. Mater. Inter.* **2015**, *7*, 22669-22677.

(56) Shi, L.; Zhang, J.; Liu, H.; Que, M.; Cai, X.; Tan, S.; Huang, L. Flower-like $Ni(OH)_2$ hybridized g-C_3N_4 for high-performance supercapacitor electrode material. *Mater. Lett.* **2015**, *145*, 150-153.

(57) Sun, J.; Wang, J.; Li, Z.; Ou, J.; Niu, L.; Wang, H.; Yang, S. Graphene-wrapped $Ni(OH)_2$ hollow spheres as novel electrode material for supercapacitors. *J. Nanosci. Nanotechno.* **2015**, *15*, 7010-7017.

(58) Li, W.; Shih, Y.; Cheng, H. Green synthesis of CNTs/$Ni(OH)_2$ nanostructures for electrochemical supercapacitors. *Chem. Phys. Lett.* **2020**, *750*, 137499.

(59) Liu, J.; Wang, Y.; Hu, R.; Munir, H. A.; Liu, H. High-performance supercapacitor electrode based on 3D rose-like beta- $Ni(OH)_2$/rGO nanohybrid. *J. Phys. Chem. Solids* **2020**, *138*, 109297.

(60) Bramhaiah, K.; Alex, C.; Singh, V. N.; John, N. S. Hybrid films of $Ni(OH)_2$ nanowall networks on reduced graphene oxide prepared at a liquid/liquid interface for oxygen evolution and supercapacitor applications. *Chemistryselect* **2019**, *4*, 2519-2528.

(61) Jiang, C.; Zhao, B.; Cheng, J.; Li, J.; Zhang, H.; Tang, Z.; Yang, J. Hydrothermal synthesis of $Ni(OH)_2$ nanoflakes on 3D graphene foam for high-performance supercapacitors. *Electrochim. Acta* **2015**, *173*, 399-407.

(62) Liu, H.; Zhang, J.; Xu, D.; Zhang, B.; Shi, L.; Huang, L.; Tan, S. In situ formation of $Ni(OH)_2$ nanoparticle on nitrogen-doped reduced graphene oxide nanosheet for high-performance supercapacitor electrode material. *Appl. Surf. Sci.* **2014**, *317*, 370-377.

(63) Ranjithkumar, R.; Arasi, S. E.; Devendran, P.; Nallamuthu, N.; Lakshmanan, P.; Sudhahar, S.; Arivarasan, A.; Kumar, M. K. Investigations and fabrication of $Ni(OH)_2$ encapsulated carbon nanotubes nanocomposites based asymmetrical hybrid electrochemical supercapacitor. *J. Energy Storage* **2020**, *32*, 101934.

(64) Wu, Q.; Wen, M.; Chen, S.; Wu, Q. Lamellar-crossing-structured $Ni(OH)_2$/CNTs/$Ni(OH)_2$ nanocomposite for electrochemical supercapacitor materials. *J. Alloy. Compd* **2015**, *646*, 990-997.

(65) Bag, S.; Raj, C. R. Layered inorganic organic-hybrid material based on reduced graphene oxide and alpha-$Ni(OH)_2$ for high performance supercapacitor electrodes. *J. Mater. Chem. A* **2014**, *2*, 17848-17856.

(66) Fu, M.; Zhu, Z.; Zhang, Z.; Zhuang, Q.; Chen, W.; Liu, Q. Microwave deposition synthesis of $Ni(OH)_2$/sorghum stalk biomass carbon electrode materials for supercapacitors. *J. Alloy. Compd* **2020**, *846*, 156376.

(67) Wang, G.; Qi, K.; Yan, Z.; Yue, L.; Ding, Y.; Li, W.; Xu, Z. Microwave hydrothermal synthesis of La decorated $Ni(OH)_2$ nanosheets for performance-enhanced hybrid supercapacitor. *Appl. Surf. Sci.* **2022**, *592*, 153293.

(68) Hu, X.; Li, J.; Wu, Q.; Zhang, Q.; Wang, X. MOF-derived $Ni(OH)_2$ nanocubes/GO for high-performance supercapacitor. *Chemistryselect* **2019**, *4*, 7922-7926.

(69) Chaitra, K.; Nagaraju, N.; Nagaraju, K. Nanocomposite of hexagonal beta-$Ni(OH)_2$/multiwalled carbon nanotubes as high performance electrode for hybrid supercapacitors. *Mater. Chem. Phys.* **2015**, *164*, 98-107.

(70) Wang, R.; Jayakumar, A.; Xu, C.; Lee, J. $Ni(OH)_2$ nanoflowers/graphene hydrogels: a new assembly for supercapacitors. *ACS Sustain. Chem. Eng.* **2016**, *4*, 3736-3742.

(71) Zeng, W.; Feng, Q.; Yuan, J. $Ni(OH)_2$/3D-rGO supercapacitor material with high specific capacitance. *Mater. Today Commun.* **2021**, *27*, 102292.

(72) Xu, L.; Chen, H.; Shu, K. $Ni(OH)_2$/rGO nanosheets constituted 3D structure for high-performance supercapacitors. *J. Sol-Gel Sci. Techn.* **2016**, *77*, 463-469.

(73) Ramesh, S.; Karuppasamy, K.; Yadav, H. M.; Lee, J.; Kim, H.; Kim, H.; Kim, J. $Ni(OH)_2$-decorated nitrogen doped mwcnt nanosheets as an efficient electrode for high performance supercapacitors. *Sci. Rep.* **2019**, *9*, 6034.

(74) Wang, H.; Shi, X.; Zhang, W.; Yao, S. One-pot hydrothermal synthesis of flower-like beta-$Ni(OH)_2$ encapsulated by reduced graphene oxide for high-performance supercapacitors. *J. Alloy. Compd* **2017**, *711*, 643-651.

(75) Chen, X.; Chen, X.; Zhang, F.; Yang, Z.; Huang, S. One-pot hydrothermal synthesis of reduced graphene oxide/carbon nanotube/alpha-$Ni(OH)_2$ composites for high performance electrochemical supercapacitor. *J. Power Sources* **2013**, *243*, 555-561.

(76) Zhang, Y.; Sun, L.; Lv, K.; Zhang, Y. One-pot synthesis of $Ni(OH)_2$ flakes embeded in highly-conductive carbon nanotube/graphene hybrid framework as high performance electrodes for supercapacitors. *Mater. Lett.* **2018**, *213*, 131-134.

(77) Zhang, H.; Zhang, X.; Zhang, D.; Sun, X.; Lin, H.; Wang, C.; Ma, Y. One-step electrophoretic deposition of reduced graphene oxide and $Ni(OH)_2$ composite films for controlled syntheses supercapacitor electrodes. *J. Phys. Chem. B* **2013**, *117*, 1616-1627.

(78) Dong, B.; Zhou, H.; Liang, J.; Zhang, L.; Gao, G.; Ding, S. One-step synthesis of free-standing alpha-$Ni(OH)_2$ nanosheets on reduced graphene oxide for high-performance supercapacitors. *Nanotechnology* **2014**, *25*, 435403.

(79) Geioushy, R. A.; Attia, S. Y.; Mohamed, S. G.; Ragab, A. A.; Fouad, O. A. Polyvinylpyrrolidone and freeze drying-assisted growth of an alpha-$Ni(OH)_2$/reduced graphene oxide hybrid structure as a superior electrode material for supercapacitors. *New J. Chem.* **2021**, *45*, 10012-10020.

(80) Li, W.; Chen, Y.; Li, F.; Zheng, W.; Yin, J.; Chen, X.; Chen, L. Preparation of amorphous detrital Ni $(OH)_2$-reduced graphene oxide composite as electrode material for supercapacitor. *Ionics* **2019**, *25*, 2401-2409.

(81) Wu, J.; Ge, F.; Li, Y. Preparation of $Ni(OH)_2$/MWCNTs composite for supercapacitor application. *Int. J. Electrochem. Sc.* **2017**, *12*, 9665-9674.

(82) Liu, Y. F.; Yuan, G. H.; Jiang, Z. H.; Yao, Z. P.; Yue, M. Preparation of $Ni(OH)_2$-graphene sheet-carbon nanotube composite as electrode material for supercapacitors. *J. Alloy. Compd* **2015**, *618*, 37-43.

(83) Cai, J.; Zhang, D.; Ding, W.; Zhu, Z.; Wang, G.; He, J.; Wang, H.; Fei, P.; Si, T. Promising rice-husk-derived carbon/$Ni(OH)_2$ composite materials as a high-performing supercapacitor electrode. *ACS Omega* **2020**, *5*, 29896-29902.

(84) Qi, Y.; Liu, Y.; Zhu, R.; Wang, Q.; Luo, Y.; Zhu, C.; Lyu, Y. Rapid synthesis of $Ni(OH)_2$/graphene nanosheets and NiO@$Ni(OH)_2$/graphene nanosheets for supercapacitor applications. *New J. Chem.* **2019**, *43*, 3091-3098.

(85) Xu, J.; Tang, M.; Hu, Z.; Hu, X.; Zhou, T.; Song, K.; Wu, J.; Cheng, J. Standing and lying $Ni(OH)_2$ nanosheets on multilayer graphene for high-performance supercapacitors. *Nanomaterials* **2021**, *11*, 1662.

(86) Shao, G.; Yao, Y.; Zhang, S.; He, P. Supercapacitor characteristic of La-doped $Ni(OH)_2$ prepared by electrode-position. *Rare Metals* **2009**, *28*, 132-136.

(87) Zhang, C.; Chen, Q.; Zhan, H. Supercapacitors based on reduced graphene oxide nanofibers supported $Ni(OH)_2$ nanoplates with enhanced electrochemical performance. *ACS Appl. Mater. Inter.* **2016**, *8*, 22977-22987.

(88) Krishnaveni, M.; Suresh, C. M.; Wu, J. J.; Asiri, A. M.; Anandan, S.; Ashokkumar, M. Synthesis of 3D marigold flower-like rGO/BN/$Ni(OH)_2$ ternary nanocomposites for supercapacitor applications. *Sustain. Energ. Fuels* **2020**, *4*, 3090-3101.

(89) Anwer, S.; Bin Ari, A.; Bharath, C.; Cao, P.; Patole, S. P.; Luo, S.; Masood, H. T.; Cantwell, W. J.; Liao, K.; Li, Q.; et al. Synthesis of joint-welded carbon nanotube foam @ $Ni(OH)_2$ nanosheet-based core-shell 3D architecture for freestanding flexible electrode for supercapacitor applications. *Adv. Mater. Interfaces* **2019**, *6*, 1900670.

(90) Bai, J.; Yan, H.; Liu, Q.; Liu, J.; Li, Z.; Bai, X.; Li, R.; Wang, J. Synthesis of layered alpha-$Ni(OH)_2$/rGO composites by exfoliation of alpha-$Ni(OH)_2$ for high-performance asymmetric supercapacitors. *Mater. Chem. Phys.* **2018**, *204*, 18-26.

(91) Jiang, C.; Zhan, B.; Li, C.; Huang, W.; Dong, X. Synthesis of three-dimensional self-standing graphene/$Ni(OH)_2$ composites for high-performance supercapacitors. *RSC Adv* **2014**, *4*, 18080-18085.

(92) Yuan, S.; Wang, X.; Lu, C.; Chen, C. The fine control of porous pompon-like Mg-incorporated alpha-$Ni(OH)_2$ for enhanced supercapacities. *Funct. Mater. Lett.* **2016**, *9*, 1650057.

(93) Wang, H.; Song, Y.; Liu, W.; Yan, L. Three dimensional $Ni(OH)_2$/rGO hydrogel as binder-free electrode for asymmetric supercapacitor. *J. Alloy. Compd.* **2018**, *735*, 2428-2435.

(94) Hussain, N.; Yang, W.; Dou, J.; Chen, Y.; Qian, Y.; Xu, L. Ultrathin mesoporous F-doped -$Ni(OH)_2$ nanosheets as an efficient electrode material for water splitting and supercapacitors. *J. Mater Chem A* **2019**, *7*, 9656-9664.

(95) Zhang, X.; Wang, H.; Shui, L.; Zhou, G.; Wang, X.; Ma, R.; Wang, J. Ultrathin $Ni(OH)_2$ layer coupling with graphene for fast electron/ion transport in supercapacitor. *Sci. China Mater.* **2021**, *64*, 339-348.
